# Active control of polariton-enabled long-range energy transfer

**DOI:** 10.1515/nanoph-2023-0677

**Published:** 2024-01-22

**Authors:** Alessio Cargioli, Maksim Lednev, Lorenzo Lavista, Andrea Camposeo, Adele Sassella, Dario Pisignano, Alessandro Tredicucci, Francisco J. Garcia-Vidal, Johannes Feist, Luana Persano

**Affiliations:** Dipartimento di Fisica “E. Fermi”, Università di Pisa, Largo B. Pontecorvo 3, I-56127 Pisa, Italy; NEST, Istituto Nanoscienze-CNR and Scuola Normale Superiore, I-56127 Pisa, Italy; Departamento de Física Teórica de la Materia Condensada and Condensed Matter Physics Center (IFIMAC), Universidad Autónoma de Madrid, E-28049 Madrid, Spain; Dipartimento di Scienza dei Materiali, Università degli Studi di Milano-Bicocca, Via Roberto Cozzi 55, I-20125 Milano, Italy; Dipartimento di Fisica “E. Fermi” and Center for Instrument Sharing (CISUP), Università di Pisa, Largo B. Pontecorvo 3, I-56127 Pisa, Italy; NEST, Istituto Nanoscienze-CNR, I-56127 Pisa, Italy

**Keywords:** molecular polaritons, polaritonic chemistry, strong light-matter coupling, organic molecules, energy transfer

## Abstract

Optical control is achieved on the excited state energy transfer between spatially separated donor and acceptor molecules, both coupled to the same optical mode of a cavity. The energy transfer occurs through the formed hybrid polaritons and can be switched on and off by means of ultraviolet and visible light. The control mechanism relies on a photochromic component used as donor, whose absorption and emission properties can be varied reversibly through light irradiation, whereas in-cavity hybridization with acceptors through polariton states enables a 6-fold enhancement of acceptor/donor contribution to the emission intensity with respect to a reference multilayer. These results pave the way for synthesizing effective gating systems for the transport of energy by light, relevant for light-harvesting and light-emitting devices, and for photovoltaic cells.

## 1 Introduction

In the strong light–matter coupling regime, photons confined within an optical cavity interact with material emitters, thus changing the fundamental physical properties of the coupled system and creating hybrid light–matter states [1], [2]. Excitations of these states are quasiparticles named polaritons, carrying features of both photons and excitons. One consequence of polariton formation is that the energy spectrum of the system changes, featuring two peaks separated, at zero cavity-transition detuning, by the Rabi splitting. The potential to modify material properties and chemistry underneath through strong light–matter coupling has stimulated enormous interest from the scientific community, both at the fundamental level and for its potential technological applications [3]–[5]. Organic materials provide relevant opportunities in this context, due to their large oscillator strengths that can lead to the achievement of large Rabi splitting values. Frenkel excitons [6] might strongly localize in organics at single-molecule level, with binding energies of the order of 1 eV [7], and Rabi splittings of hundreds of meV might enable the observation of macroscopic quantum phenomena at room temperature. In this framework, some remarkable achievements include room temperature Bose–Einstein condensation [8], polariton lasing [9], [10], tunable third harmonic generation [11], and increased efficiency in organic photovoltaics (OPVs) [12]. Indeed, one of the main challenges in OPVs is the improvement of the power conversion efficiencies (PCE) [13], [14], that suffers from the relatively large non-radiative decay rates and the typically incoherent, diffusive nature of exciton transport. The delocalization of polaritons in the collective strong coupling regime, which originates from the photonic component, has the potential to enhance energy transfer efficiencies overcoming low exciton transport and charge carrier mobility, thus effectively leading to an improvement of the overall efficiency of light harvesting [12]. The long-range energy transfer offered by polariton states already led to a promising outlook for the enhancement of the PCE [15].

In conventional Förster-type energy transfer processes, energy transport is based on exciton dipole-dipole interactions between a donor and an acceptor molecule, with a low effective range of a few nm [7]. This usually requires physical blending of different molecular components to enable energy transport. Instead, in the strong coupling regime the quantum-mechanical entanglement of the donor and acceptor molecules within the polaritonic states enables a new energy transport mechanism that is no longer dependent on the spatial distance [16], [17]. Several reports indicate that mixed exciton–polariton states serve as fast pathways for the energy transfer from the donor molecules to the acceptor ones [18]–[20] and, consequently the spatial range of transfer has been extended from 10 nm [21] to a few micrometers [16], [22]. These results have been obtained by physically separating the donor and acceptor molecules by embedding them in layered systems with a transparent spacer [23]. In general, long-range transfer is not only limited to molecular systems, but has been also demonstrated with carbon nanotubes excitons [24], [25] and vibrational excitations [26]. However, such systems are basically static. While the coupling parameters are traditionally permanently defined by a given cavity design (i.e. layer composition, thickness, and topology), dynamic systems where external stimuli might activate or deactivate polariton states [27]–[30] would open much more exciting perspectives for precisely controlling energy flows in intelligent resonant photonics.

Here we propose a new class of optical cavities based on a photogateable donor-acceptor system, in which UV-light driven photoisomerization directly affects the energy transfer mechanism. A microcavity architecture is developed consisting of two different dye layers, sequentially deposited from orthogonal solvents to form the donor-acceptor system. UV light irradiation activates the photoisomerization process of the donor, thus controlling the concentration of transfer-available components in the cavity. As the concentration increases, polariton states start involving the donor and the energy transfer process to the acceptor is activated. Furthermore, irradiation with visible light switches back the energy levels to the initial uncoupled conditions, thus deactivating the polariton-assisted energy transfer process. The capability to control complex energy flows in photonic devices by means of external light provides additional functionalities and opportunities in light harvesting based on strong light–matter coupling.

## 2 Results and discussion

**Figure 1:**
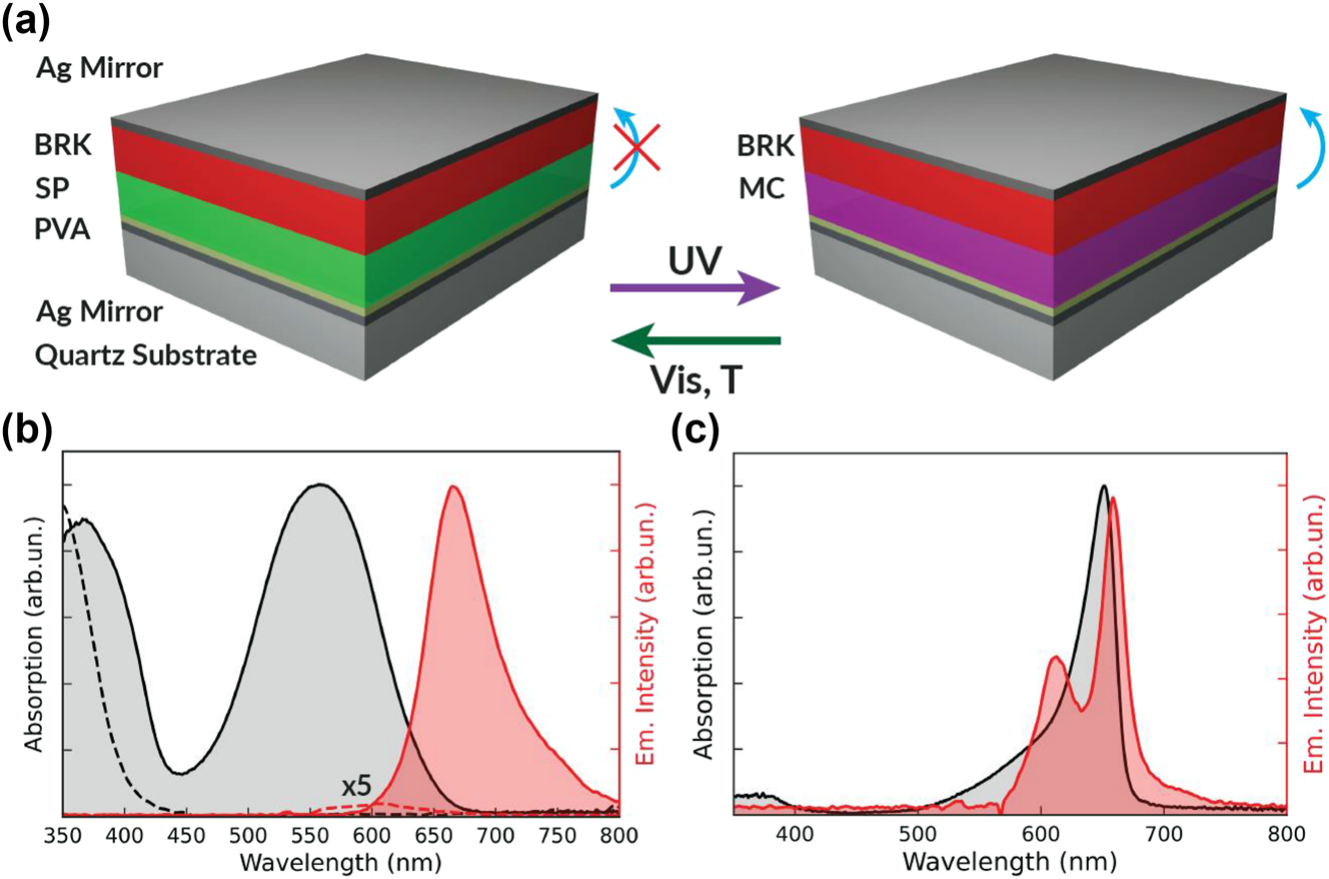
Device architecture and molecular system. (a) Schematics of the cavity before and after photochromic donor conversion. The donor molecule, initially transparent in the visible range in its SP form, is converted to a colored MC form by irradiation with UV light (violet arrow), whereas the back-conversion can occur by irradiation with green light (green arrow) or by thermal relaxation. The vertical bent arrows represent the donor-acceptor energy transfer in the two configurations. (b) Absorption spectrum of a PMMA film with SP (black dashed lines) and MC (black continuous line) and corresponding PL spectrum of the SP (×5 intensity, red dashed line) and MC form (converted by UV exposure for 5 s, red continuous line). (c) Absorption (black line) and emission (red line) of a film of PVA doped with BRK. The excitation wavelength for the emission measurements is 532 nm.

The microcavity architecture, schematised in Figure 1a, consists of two Ag films as mirrors, sandwiching two spatially separated photoactive layers.

The absorption and emission of the donor layer, which is based on the photochromic 1,3,3-trimethylindolino-6′-nitrobenzopyrylospiran (SP) [28] in a host matrix of poly(methyl methacrylate) (PMMA), are reversibly varied by UV and green light irradiation. The SP film is transparent in the 450–800 nm range (Figure 1b) and exhibits a highly uniform morphology (root mean square roughness = 0.3 nm, Figure S1a and b). Upon irradiation with UV light (*λ*_UV_ = 365 nm), SP converts to merocyanine (MC). For each value of the duration of the UV exposure, a mixture of SP and MC is obtained, with relative content depending on the specific irradiation conditions and only the MC component being coupled to the cavity mode. MC features a strong absorption peaked at 554 nm, while its photoluminescence (PL), measured with a pump laser at 532 nm, is peaked at 663 nm (Figure 1b). The PL from the PMMA-SP film under the same excitation conditions features only a very weak peak at 600 nm (Figure 1b), in agreement with previous reports [31].

The controlled photochromic conversion is exploited to activate/deactivate the coupling to the microcavity, and the resulting excited state energy transfer to the acceptor molecules. The length of the active region is chosen to have the second-order resonant mode at about 620 nm at normal incidence (inter-mirror distance = 355 nm), a configuration that is expected to enhance the light–matter coupling for both the donor and acceptor molecules [16], [23] (Figure S2), and additionally minimizes interface effects between the two layers as the field has a local minimum there. The J-aggregate [32] form of 3,3′-Bis(3-sulfopropyl)-4,5:4′,5′-dibenzo-9-ethylthiacarbocyanine betaine thiethylammonium salt (BRK) [16], [19] is used as acceptor, embedded in a host matrix of polyvinyl alcohol (PVA). BRK absorbs at 655 nm, whereas its emission shows peaks at 612 nm and 659 nm, respectively (Figure 1c). The most intense peak is attributed to fully formed J-aggregates, while the smaller and blue-shifted one is traceable to some BRK molecules which do not aggregate in the fabrication process (see Supplementary Information–SI, Figure S3). While contributing to BRK absorption broadening, non-aggregated molecules do not interfere severely with the polariton formation since their number, and thus their coupling to the cavity field, is sufficiently small compared to the fully formed aggregates. The kinetics of the SP-to-MC photochromic conversion upon exposure to 365 nm light is shown in Figure S4. Further details on microcavity fabrication are reported in the Methods, Section 2 of SI and Figure S5.

**Figure 2:**
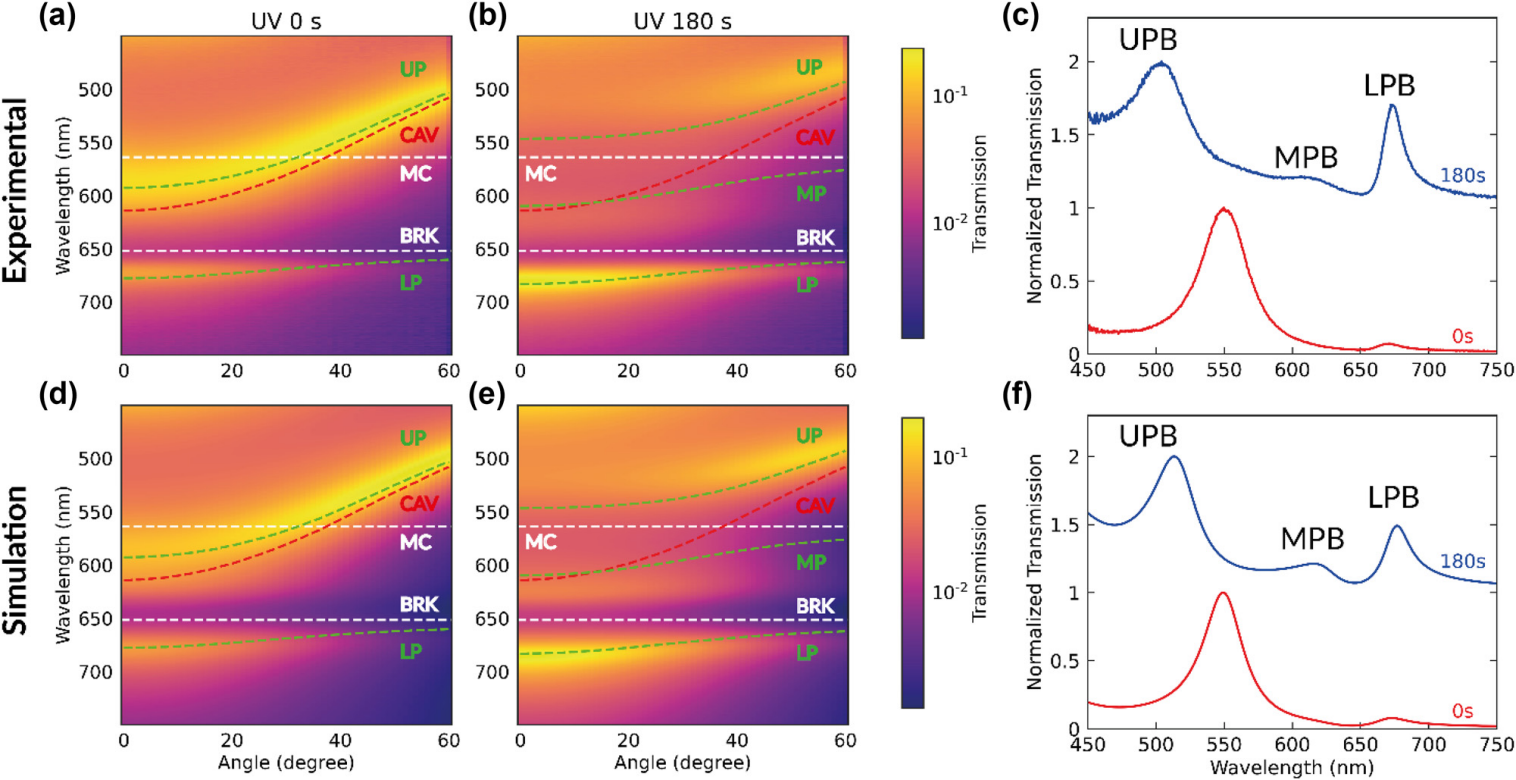
Angle-resolved transmission. (a, b) Experimental angle-resolved transmission maps at different UV exposure times, and transmission spectra (c) before (red line) and after (blue line) irradiation at the anticrossing angle (37°). (d–f) Corresponding simulated maps and transmission spectra. In the colorscale, unity stands for total transmission. In each colormap the bare cavity mode (red dashed line), the MC excitonic transition (upper white dashed line) and the BRK excitonic transition (lower white dashed line) are also reported. The green dashed lines are the result of a fit using the coupled oscillators model [30]. The transmission spectra shown in (c) and (f) are divided by their maximum value, respectively, and vertically shifted for better clarity.

**Figure 3:**
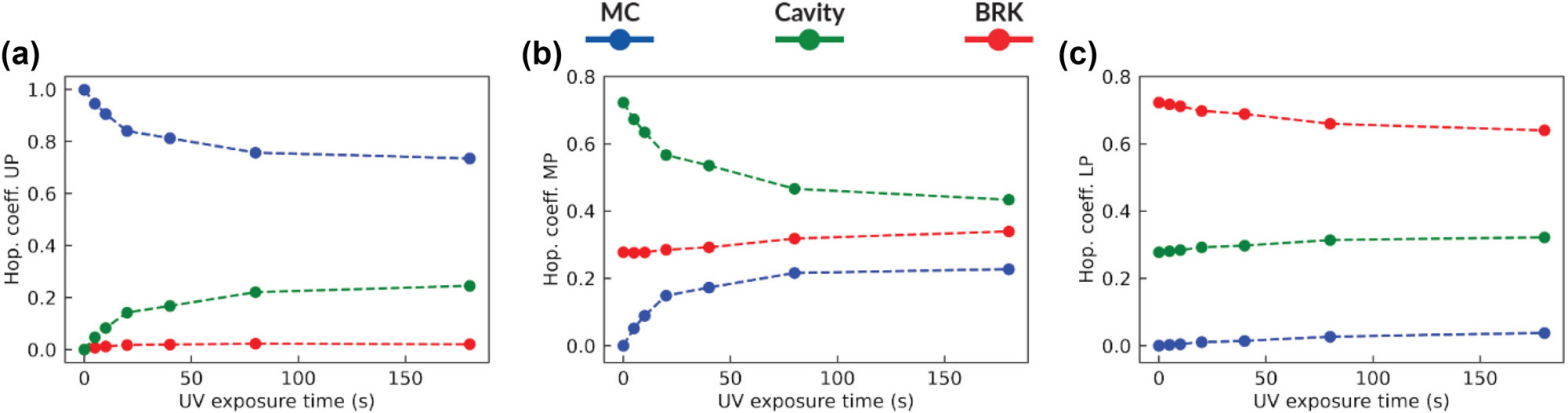
Hopfield coefficients. Hopfield coefficients at 0° of upper (a), middle (b), and lower (c) polariton branches as a function of the UV exposure time.

The optical transmission properties of the microcavity before and after 180 s of UV exposure are illustrated in Figure 2. Optical transmission measurements at intermediate UV exposure times are reported in Figure S6. The experimental data are compared with simulated transmission maps, computed through a transfer matrix approach [33]. To this aim, the wavelength dispersion of the complex refractive index of PMMA-MC and PVA-BRK are derived from the optical transmission measurements performed on reference first-order cavities embedding either BRK-doped PVA or MC-doped PMMA, respectively (details in Section 4 of SI, Figures S7 and 8). In pristine devices (UV exposure time = 0 s), only the BRK aggregates couple to the cavity field at visible wavelengths. Since the BRK absorption is off resonance from the cavity dispersion, we observe only a slight shift with respect to the uncoupled bands (the resulting light–matter coupling constant for BRK is *g*_BRK_ = 117 meV). Once MC is introduced in the system by means of UV irradiation, two exciton species can participate in the polariton formation and three polaritonic branches appear (Figure 2), i.e., the upper polariton branch (UPB) at about 503 nm, the middle polariton branch (MPB) at 612 nm and the lower polariton branch (LPB) at 675 nm (all wavelengths at normal incidence). A fit of the polaritonic dispersions is also performed using a coupled oscillator model [34], which allows the Hopfield coefficients to be retrieved for each polariton branch (see Section 5 and Figures S9–11). The results of this analysis at 0° are shown in Figure 3. As expected [16], [19], [34], the UPB (LPB) is mostly composed of a photonic component and a donor (acceptor) molecular component, while the MPB has a more balanced nature involving the three components. By increasing the exposure time to UV light, the Hopfield coefficient of MC is increased up to about 0.2 for the MPB, while its cavity component is lowered down to about 0.4. The MPB highlights the possibility to control the degree of hybridization between the donor and acceptor molecules by an external light signal. We also mention that in between the polaritonic branches visible in the transmission spectra, the system also contains “dark states” or “exciton reservoirs” corresponding to the excitonic transitions of the donor and acceptor molecules that are not coupled to the cavity field.

The light–matter coupling strength is known to depend on the square root of number of emitters interacting with the cavity field [35], [36]. Since we are actively changing the concentration of donors available to energy transfer (MC), we expect the light–matter coupling constant to increase with UV-exposure time (*t*_exp_). Through the coupled oscillators model (see Section 2 and SI for details), we extract the parameter *g*_MC_(*t*_exp_) from the transmission measurements. As shown in Figure S12, an increase of *g*_MC_ up to 124 meV is found after 180 s of UV irradiation.

**Figure 4:**
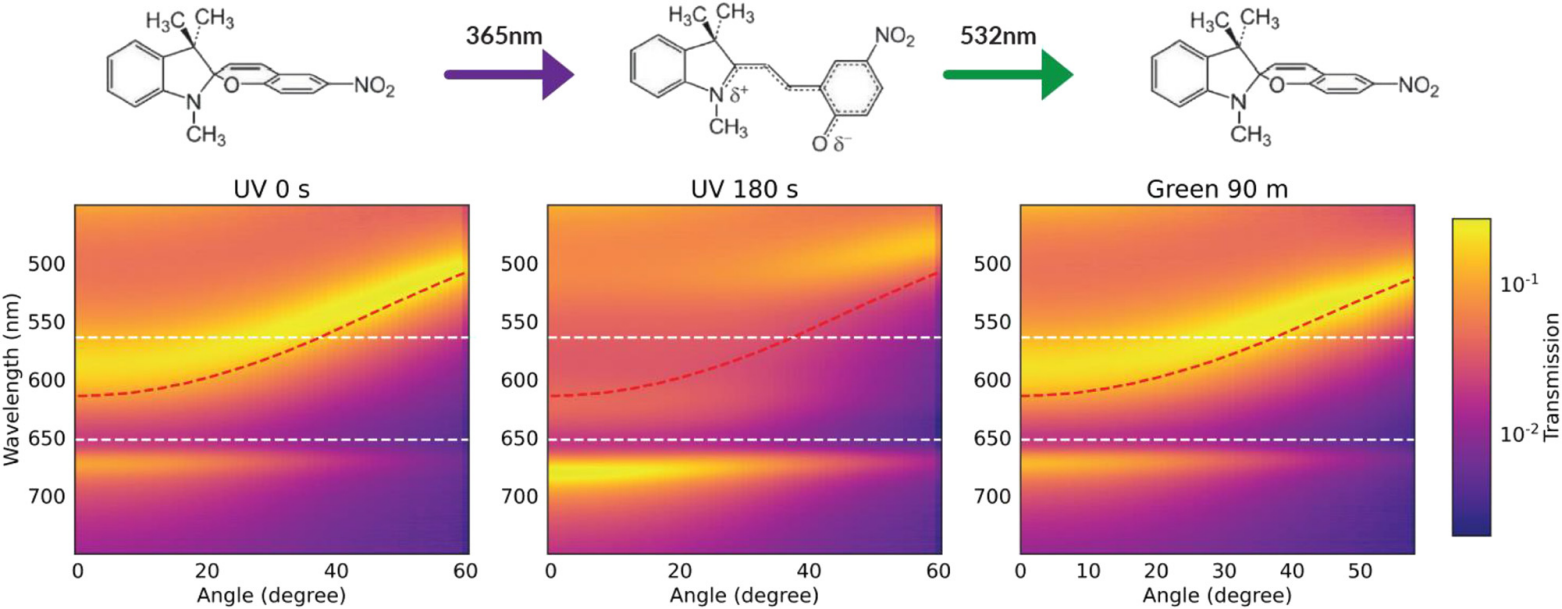
Polariton switching. Angle-resolved transmission measurements as a function of UV and green light exposure time. The photochromic conversion from SP to MC and back to SP is also schematically displayed. In each colormap, the bare cavity mode (red dashed line), the MC excitonic transition (upper white dashed line) and the BRK excitonic transition (lower white dashed line) are also reported.

The system can be back-switched [37] by irradiation with green laser light (532 nm, intensity ∼275 mW cm^−2^), which reverses the SP-MC photoisomerization (Figure 4).

The transmission maps for various times of green light exposure are reported in Figures S13 and 14. Ultimately obtained bands are largely comparable to those of the pristine device, with minor changes of signal intensity and broadening of the photonic mode most likely due to the residual MC component. The complete set of polariton branches can be observed in the system for up to four consecutive UV-green irradiation cycles (Figure S15). Fatigue effects, attributed to photo-oxidation [38], photoisomerization towards poorly back-converting forms [39], or formation of MC aggregates [40], then lead to MPB suppression. Lack of photoconversion is found for ten or more irradiation cycles in the systems investigated here. Various strategies have been developed to reduce fatigue in SP/MC compounds, including covalent attachment of modified SP to polymer films [41] and embedment of sulfonated SP in silica matrix [42].

**Figure 5:**
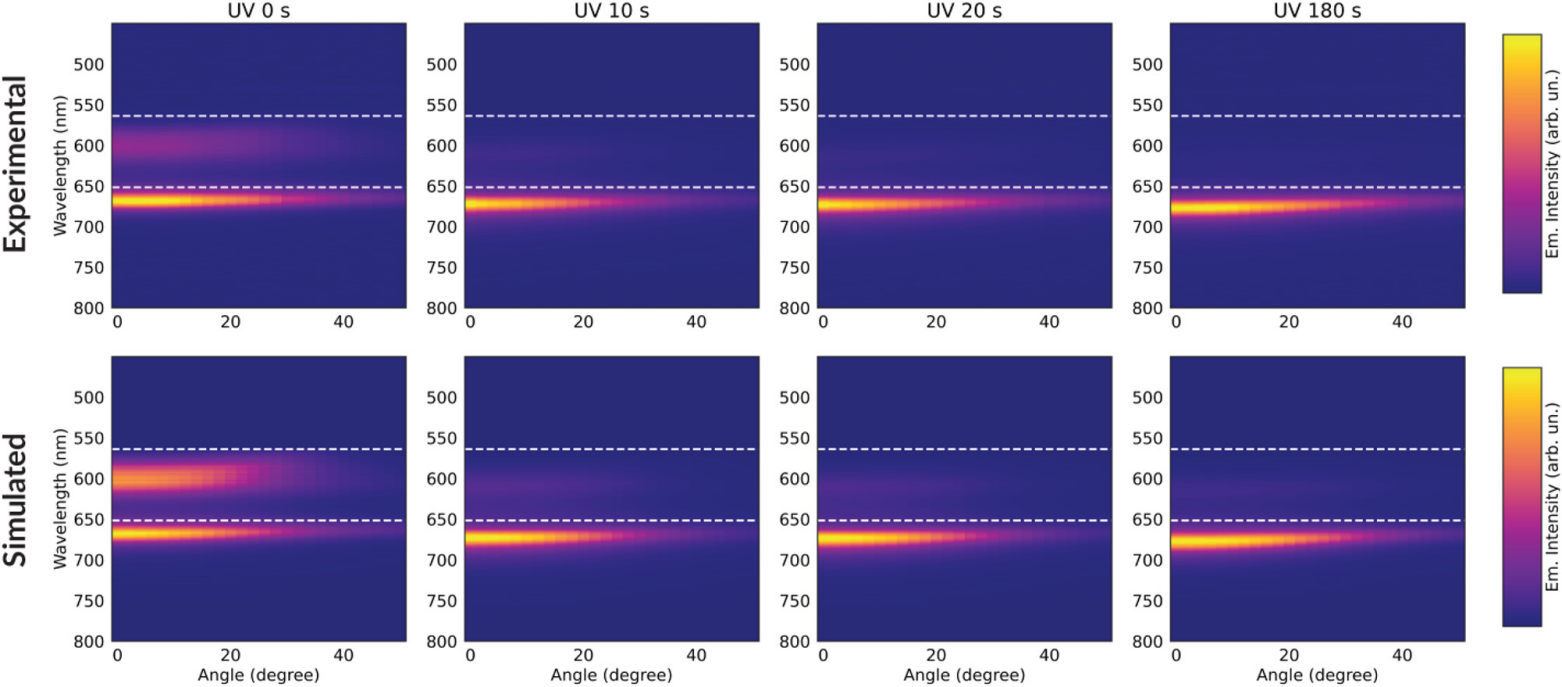
Angle-resolved PL. Experimental and simulated angle-resolved emission maps of the cavity after UV exposure time of 0 s, 10 s, 20 s and 180 s (from left to right) and then excited by a 532 nm pump (<1 min). White dashed lines show the spectral wavelengths of donor and acceptor absorption peaks.

The angle-resolved emission from cavities after different UV exposure times and then shortly excited by a 532 nm laser are shown in Figure 5. The corresponding emission spectra are reported in Figure S16. The emission spectrum of the cavity before irradiation with UV light shows two bands peaked at 600 nm and 668–671 nm, respectively. As soon as the UV light is switched on, the upper band intensity decreases and finally disappears while the lower one red-shifts and its intensity increases. In agreement with the transmission data, a back-conversion of the PL signal is found upon longer (5–90 min) irradiation with green light (Figures S17 and 18).

**Figure 6:**
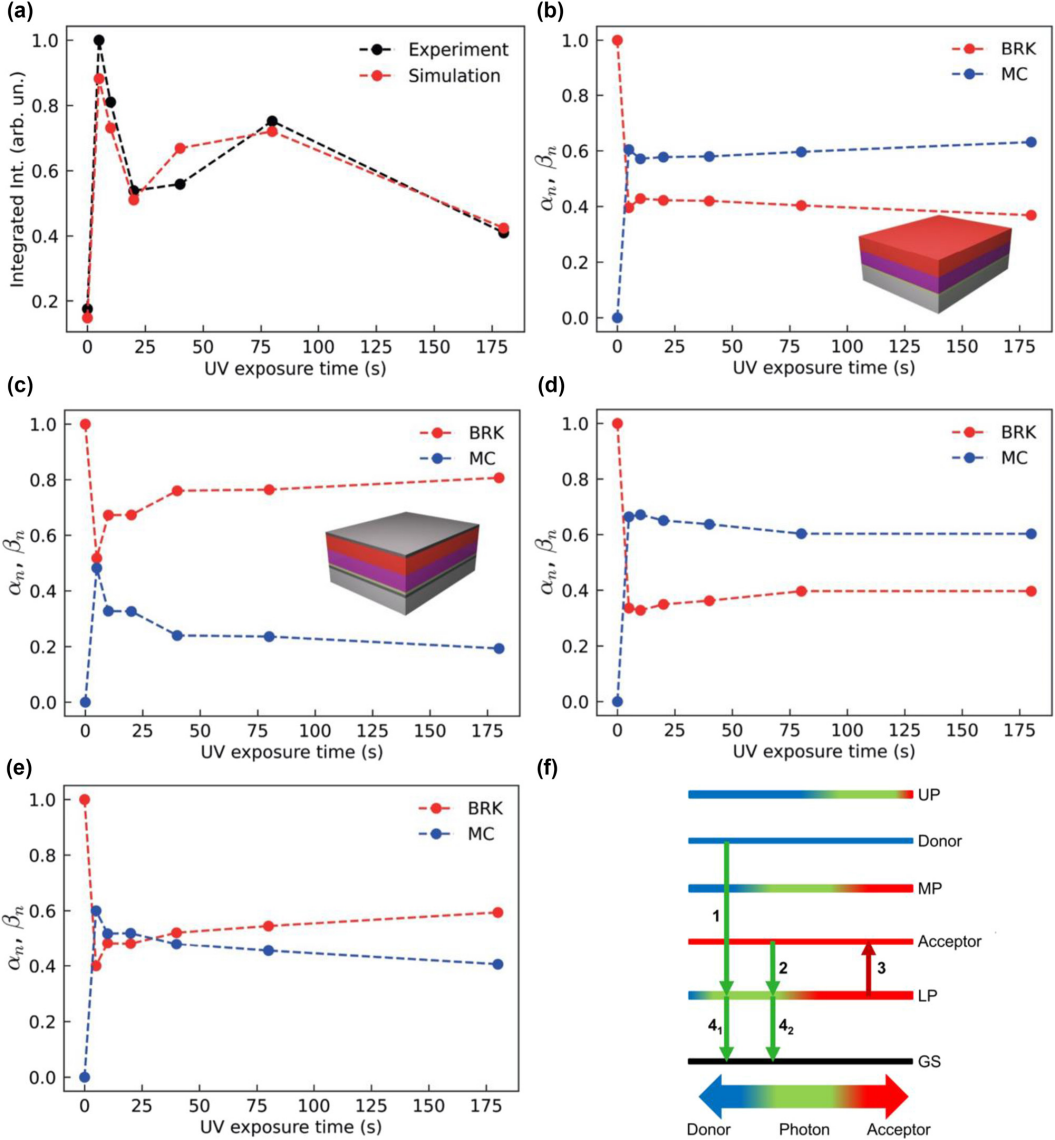
Modelling the light-controlled energy transfer mechanism. (a) Simulated (red circles) and measured (black circles) integrated emission intensity of the LP at 0° as a function of the UV exposure time. Experimental and simulated data are divided by the maximum value of the experimental data. (b–c) Normalised weight coefficients, *α*_*n*_ (red circles) and *β*_*n*_ (blue circles), as a function of UV exposure time for the MC/BRK system outside and inside the cavity, respectively. (d–e) *α*_*n*_ (red circles) and *β*_*n*_ (blue circles) for the off-resonance MC/BRK system outside and inside the cavity, respectively. (f) Schematic representation of the relevant energy levels and of the emission decay pathway for the cavity analyzed in (a–c).

The observed emission spectrum can be rationalized from a model in which the emission from the molecular reservoirs to the outside is calculated using transfer matrix theory, which can be conceptually understood as the polaritonic modes behaving as a filter for the emission from the molecular reservoir. Although the emitted light is transmitted from within the cavity to the outside, the relevant filter function can be well-approximated as the conventional cavity transmission function (see Section 9 and Figure S19 in SI). This does not imply that the cavity and polariton formation has no effect apart from acting as a cavity filter, since the cavity and polariton modes furthermore act to mediate efficient donor-acceptor energy transfer as discussed in the following. Thus, we represent the emission as the PL signal of the bare molecules modulated by the cavity transmission:

(1) $$\begin{array}{ll} I_{\text{cav}}\left(\omega ,\theta ,t_{\exp}\right) & =T_{\text{cav}}\left(\omega ,\theta ,t_{\exp}\right)\left[\alpha \left(t_{\exp}\right)I_{\text{BRK}}\left(\omega \right)\right.\\ & \;\left.+\beta \left(t_{\exp}\right)I_{\text{MC}}\left(\omega ,t_{\exp}\right)\right] \end{array}$$

where *T*_cav_ is the cavity transmission of the hybrid system, *I*_BRK_, *I*_MC_ are the emission intensities of the molecules as measured outside the cavity, and *α*, *β* are phenomenological weight coefficients. The coefficients effectively represent the contributions of both molecular species to the emission of the hybrid system. For the simulation of the emission maps, we fit *I*_cav_ (Eq. (1)) to the experimental PL intensity from the cavity, using the weight coefficients *α*, *β* as free parameters. This approach for the simulation of the emission properties of the cavity is equivalent to a rate equation model, which has been successfully applied for the interpretation of the emission measurements in similar systems [34], [43], [44] under the assumption that radiative pumping [45] is the dominant population mechanism for the LPB, i.e., the vibrational scattering from the acceptor excitonic reservoir is negligible (see Section 10 and Figures S20–21 of SI for details). In our hybrid system, the used approximation is valid since radiative pumping occurs not only from the acceptor reservoir, but also from the donor one, due to the fact that the MC emission has a significant overlap with the LPB. The results of the simulations are shown in the bottom of Figure 5. As an example of validity of the model, we report in Figure 6a the comparison between the measured and simulated integrated emission intensity of the LP at a fixed angle (in this case 0°) as a function of the UV exposure time.

The simulated data reproduces the experimental measurements well. They demonstrate that the increase in the number of MC molecules not only dramatically changes the absorption properties of the system but also its emission, with a direct impact on the energy transport from donor to acceptor molecule. Thus, we can fully interpret the emission spectra dynamics of the hybrid system under UV illumination. At 0 s of UV irradiation, the upper band is attributed to the emission of non-aggregated BRK molecules which leaks through the cavity mode slightly modified by interaction with BRK molecules. After the start of the UV illumination the hybrid states shift due to an increase in the donor coupling strength. This, in turn, leads to a quenching of the upper emission band, since the emission peak of the BRK molecules is not in resonance with the transmission bands of the hybrid system. For the lower emission band the situation is completely different, since for the whole range of UV irradiation times of the measurements the LPB remains in resonance with the main emission peaks of the donor and acceptor molecules. Once the donor is introduced with varied amounts in the system by UV irradiation, the emission of the multilayer outside and inside the cavity become remarkably different (Figures S22 and 23): outside the cavity both donor and acceptor molecules contribute to a broad and spectrally-stable overall emission, whereas for the cavity a red-shift (∼8 nm) of the emission peak is found upon increasing the UV exposure time. It is worth noting that also control cavities involving either only the acceptor (Figures S24–26) or only the donor (Figures S27–29) show a substantially different behavior. The transmission and emission of the acceptor-only cavity are almost unaffected by the UV irradiation. Instead, in the donor-only cavity polariton bands still vary upon UV irradiation due to the photo-induced SP-to-MC conversion, with emission occurring at a different wavelength range with respect to the donor–acceptor cavity (i.e., at 648–658 nm, related to the specific LP formed in presence of the unique PMMA-SP/MC layer).

Our approach allows us not only to clearly understand the origin of the observed experimental PL features, but also to track the contributions of both molecular species to the emission through the weight coefficients in Eq. (1). Following an analogous procedure, we find the weight coefficients corresponding to BRK and MC molecules for emission of the multilayer outside the cavity (for details see SI Section 14, Figure S30). The comparison between the normalised weight coefficients for the donor and acceptor molecules placed outside and inside the cavity is reported in Figure 6b and c, respectively (emission spectra for the PMMA-MC/PVA-BRK multilayer without cavity are shown in Figure S23a). The normalised weight coefficients are defined as $\alpha_{n}=\frac{\alpha }{\alpha +\beta }$ and $\beta_{n}=\frac{\beta }{\alpha +\beta }$ , corresponding to the relative fractions of emission arising from the BRK and MC molecules, respectively, which cannot be derived by simply comparing the integrated emission intensities of the cavity and reference multilayer (Figure S23b). Moreover, the normalisation of the coefficients is necessary to properly compare the emission properties of the multilayer outside and inside the cavity, and to rule out any dependence on the excitation efficiency which can significantly change throughout the experiments in the cavity due to the shift of the polaritonic states (see Sections 14 and 15 of SI). While in the transmission measurements, the polariton bands reach their final positions only when the reaction reaches a steady state, the emission becomes stable much more quickly, after just a few seconds of irradiation. At *t* = 0, emission is mainly from BRK. In the first few seconds of UV irradiation and donor photoisomerization, the relative amount of the BRK-related emission fraction, *α*_*n*_, is strongly reduced as a consequence of the sudden rise of MC concentration. Without the cavity (Figure 6b) this effect is very pronounced and slowly continues afterwards, consistent with the progressive achievement of the photostationary state, until *β*_*n*_ ∼ 1.5 *α*_*n*_.

On the other hand, inside the cavity, the roles are reversed and at *t*_exp_ > 5 s the BRK contribution rises and achieves a value almost 4 times higher than the MC, due to the presence of cavity-enhanced energy transfer. We additionally realize an off-resonant cavity, where the second-order resonant mode is red shifted (772 nm) by increasing the thicknesses of the donor and acceptor layers (see Section 2 for details). By doing so, the cavity mode is out of resonance from the excitonic states of the molecular species. The resulting measured angle-resolved transmission and PL spectra are reported in Section 16 of the SI (Figures S31–34), while the calculated normalised weight coefficients for a donor/acceptor multilayer out of the cavity and inside the cavity are shown in Figure 6d and e, respectively. For the reference multilayers out of cavity, the contribution to emission from MC and BRK are similar for resonant and non-resonant configurations (Figure 6b–d). Interestingly, in the off-resonance cavity, the BRK and MC almost equally contribute to the overall emission up to about 40 s of UV exposure, whereas at *t*_exp_ > 40 s the BRK contribution slightly increases reaching a value which is significantly lower than in the resonant case (*α*_*n*_ ∼ 1.5 *β*_*n*_).

We explain this behavior through the level scheme in Figure 6f. Firstly, the green laser pumps both the donor and acceptor excitonic reservoirs, then the molecules emit into the lower polariton state through its photonic component (radiative pumping mechanism corresponding to arrows 1 and 2 in the scheme). The lower polaritonic state has two main loss mechanisms: the first is radiative decay through the cavity mirrors (arrows 4_1_ and 4_2_ for light originating from the donor and acceptor reservoirs, respectively, which can be distinguished with the fitting procedure discussed above, and which are characterized by the coefficients *β* and *α*, respectively), which occurs on femtosecond timescales, while the second one is non-radiative decay to the acceptor excitonic reservoir (arrow 3), with efficiency proportional to acceptor fraction in LP. While this process is usually expected to be slower than radiative decay [34], [43], [44], [46], transfer matrix calculations of the relative absorption and emission fractions for light emitted by the donor molecules indicate that this process is actually comparably efficient to radiative decay in the current setup (see Section 10, Figure S20), which can be attributed to the relatively large overlap between the acceptor absorption spectrum and the lower polariton [46]. Its appearance significantly affects the excitation transfer pathways due to pumping of the acceptor excitonic reservoir. In particular, it provides transfer of energy from the donor reservoir to the acceptor one through the lower polaritonic state. Thus, the lower polaritonic state in our experimental setup serves as an intermediate state for energy transfer between the donor and acceptor, and is responsible for the redistribution of the donor and acceptor contributions to the emission (Figure 6b and c). Overall, in the resonant cavity the fraction of the emission due to the acceptor molecules with respect to the donor ones is enhanced by a factor of 6 compared to the bare donor/acceptor multilayer. By contrast, in the off-resonant cavity the redistribution between donor and acceptor is weakened because of the reduced efficiency of (i) the radiative pumping from the donor excitonic reservoir to the LP state (arrow 1 in Figure 6f) due to a decrease of the overlap between the LP dispersion and MC emission band and, (ii) the non-radiative relaxation of lower polaritons to the acceptor excitonic reservoir (arrow 3 in Figure 6f) since the efficiency of this channel is proportional to the acceptor fraction in the LP, which is largely decreased. Indeed, for the off-resonant case, the LP consists mostly of the photonic part (see the calculated Hopfield coefficient of the LP in Figure S35).

In conclusion, we have demonstrated the possibility of controlling the polariton formation between two different molecules via external optical gating in a donor–acceptor system. This is achieved by embedding a photo-active multilayer in an optical microcavity, in which one of the layers (the donor one) features reversible photochromic properties upon UV and visible light irradiation. These architectures enable the possibility to control the energy transport between the spatially separated species by light. Engineering externally controllable, intelligent photonic systems which could present long range energy transport might open a new way of approaching light-harvesting, light-emitting, and OPV devices and integrated platforms.

## 3 Methods

### 3.1 Microcavity fabrication

PMMA and SP with a 1:1 weight:weight (w:w) ratio are dissolved in toluene, while PVA and BRK (10:1 w:w ratio) are dissolved in a mixture of deionized water and methanol (1:1 volume ratio). Microcavities are fabricated by thermal evaporation of a 25 nm-thick Ag mirror on top of a quartz substrate (1 × 1 cm^2^) by using an MBRAUN MB-ProVap 4G system. Afterwards, the active organic multilayer is deposited onto the Ag mirror by spin coating a PVA buffer layer (thickness: 25 nm) as a first step, followed by a PMMA-SP layer (thickness: 150 nm) and a PVA-BRK layer (thickness: 180 nm). Finally, a 25-nm thick top Ag mirror is evaporated onto the PVA-BRK layer (SI, Figure S5). The thicknesses of the active organic layers in the control cavities are: donor-only cavity (see Section 13 of SI), PVA (25 nm), PMMA-SP (150 nm), PVA (180 nm); acceptor-only microcavity (see Section 12 of SI), PVA (25 nm), PMMA (150 nm), BRK-PVA (180 nm); off-resonant cavity (see Section 16 of SI), PVA (25 nm), PMMA-SP (225 nm), PVA-BRK (240 nm). The reference PVA/PMMA-SP/PVA-BRK multilayers are deposited by spin coating on top of a quartz substrate. The thicknesses of the Ag and organic layers are measured by using a stylus profilometer (DektakXT, Bruker). The surface morphology of PMMA-SP films is investigated in PeakForce tapping mode by using a probe with a nominal spring constant of 0.4 N m^−1^ (Bruker, USA) on a Bruker Dimension Icon system, equipped with a Nanoscope V controller. More details about the fabrication are reported in the SI.

### 3.2 Optical characterization and light switching

The absorption and transmission spectra of the Ag, PVA-BRK and PMMA-SP/MC layers are measured by using a spectrophotometer (Lambda950, Perkin Elmer). Spectroscopic ellipsometry measurements are performed on PVA-BRK films spin coated on a Silicon/silicon oxide substrate using the V-VASE ellipsometer (J. A. Woollam Co.) in the spectral range from 300 to 800 nm and with three angles of incidence, 70°, 75°, and 80°. The fitting procedure is carried out by means of the software WVASE32, considering a multiple-oscillator model. PL spectra of the active layers are measured by exciting the samples with a 532 nm diode-pumped solid-state laser and analyzing the emission by using a fiber-coupled monochromator (FLAME, Ocean Optics). Photochromic conversion experiments are carried out by irradiating the whole surface of the samples with a UV light emitting diode (LED, mod. M365LP1, Thorlabs, emission peaked at about 365 nm) for the conversion of SP in MC, while the back-switching is achieved by illuminating the samples with the 532 nm laser.

### 3.3 Angular transmission and PL measurements

Angle-resolved transmission measurements are performed by using the output beam of a broadband lamp (DH-2000, Ocean Optics) focused onto the sample (diameter of the spot about 0.5 mm at normal incidence). The spectrum of the lamp in air is taken as reference. A polarizer is positioned along the optical path to control the polarization of the incident light beam. The sample is placed on a holder mounted on one of two concentric rotation stages, used for varying the angle of the sample with respect to the incident beam and the angle of collection of the optics, respectively. For angular transmission measurements, the collection optics (composed by a lens system and an optical fiber) is positioned along the axis of the incident beam, while the sample is rotated. The light collected by the lens system and the optical fiber is directed to the monochromator for spectral analysis. For angular PL measurements the sample position is fixed, while the collection optics is rotated. The samples (both cavities and reference films) are excited by the 532 nm laser, impinging on the sample with an incidence angle of about 5° (spot size about 0.5 mm). The PL of the samples is collected by the lens system and the optical fiber positioned on the rotation stage, and a long-pass filter (cut-off wavelength at 550 nm) is used to attenuate the light of the excitation laser. In a typical measurement, PL angular spectra are collected with a step of 2°, while the angle of collection of the PL is about 2.5°. The excitation intensity and the exposure time of the green light during PL measurements were reduced to 19 mW/cm^2^ and 30 s, respectively, not to affect the MC to SP back-switching.

### 3.4 Modelling

For the simulation of the angle-resolved transmission spectra, TMM is used [47]. Firstly, in order to extract the refractive indices of the active layers (PVA-BRK and PMMA-SP(MC)) we carry out TMM calculations for first-order cavities containing only a PVA-BRK layer and only a PMMA-SP(MC) layer, respectively (details in SI, Section 4). Then, the full multilayer cavity with both donor and acceptor layers is considered. Using TMM, we fit the simulated spectra to the experimental ones for exposure times *t*_exp_ = 0*,* 5*,* 10*,* 20*,* 40*,* 80*,* 180 s, where the fit parameters are the thicknesses of the layers and the time-dependent dielectric permittivity of the PMMA-SP(MC) layer. The latter is modelled as $\varepsilon_{\text{donor}}\left(\omega ,t_{\exp}\right)=\gamma \left(t_{\exp}\right)\varepsilon_{\text{MC}}\left(\omega \right)+\left[1-\gamma \left(t_{\exp}\right)\right]\varepsilon_{\text{SP}}\left(\omega \right)$ , where *ɛ*_MC_, *ɛ*_SP,_ and *ɛ*_donor_ are the dielectric permittivity functions for PMMA films containing MC, SP, and their mixture, while *γ* is related to the fraction of MC molecules in the SP/MC mixture. The angle-resolved transmission spectra are also analyzed in order to obtain Hopfield coefficients. Using a coupled-oscillators model (see Section 5 of SI), we fit solutions of the model to experimentally observed transmission peaks, which allow for extraction of the coupling between the cavity and the excitonic transitions of the emitters.

## Supplementary Material

Supplementary Material Details
