# Supplementary material for: Active control of polariton-enabled long-range energy transfer
Source: Nanophotonics. 2024 Jan 22;13(14):2541–51. doi: 10.1515/nanoph-2023-0677 (PMC11147494; doi:10.1515/nanoph-2023-0677)
Supplement: Supplementary file 1 — Supplementary Material Details [file j_nanoph-2023-0677_suppl_001.pdf]

Supplementary Information

Active Control of Polariton-Enabled Long-Range Energy Transfer

A. Cargioli[1,2,*,#], M. Lednev[3,#], L. Lavista[1,2,#], A. Camposeo[2], A. Sassella[4], D. Pisignano[2,5], A. Tredicucci[2,5], F. J. Garcia-Vidal[3], J. Feist[3], and L. Persano[2,†]

[1]Dipartimento di Fisica "E. Fermi", Università di Pisa, Largo B. Pontecorvo 3, I-56127 Pisa, Italy

[2]NEST, Istituto Nanoscienze-CNR and Scuola Normale Superiore, I-56127 Pisa, Italy

[3]Departamento de Física Teórica de la Materia Condensada and Condensed Matter Physics Center (IFIMAC), Universidad Autónoma de Madrid, E-28049 Madrid, Spain

[4]Dipartimento di Scienza dei Materiali, Università degli Studi di Milano-Bicocca, Via Roberto Cozzi 55, I-20125 Milano, Italy

[5]Dipartimento di Fisica "E. Fermi" and Center for Instrument Sharing (CISUP), Università di Pisa, Largo B. Pontecorvo 3, I-56127 Pisa, Italy

[*]Contact Email (present address): acargioli@phys.ethz.ch

[†]Contact Email: luana.persano@nano.cnr.it

[#]These authors contributed equally to this work

1 Absorption and emission properties of donor and acceptor molecules

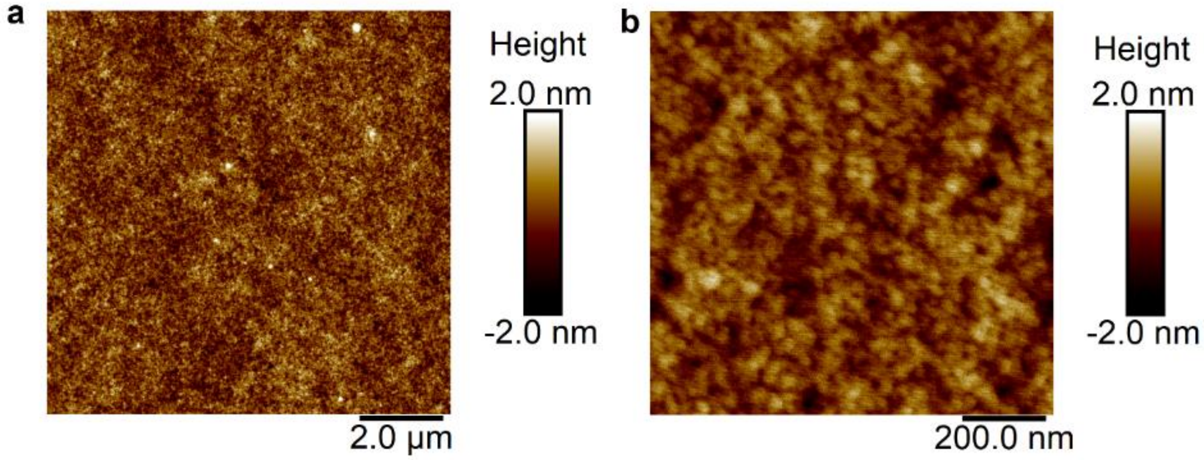

Fig. S1: a,**b**. Atomic force microscopy images, at two magnifications, of a PMMA-SP control film deposited on a PVA buffer layer. PVA is in turn deposited on a silver-coated silicon substrate. The average roughness (root mean square) is about 0.3 nm, as calculated by averaging 10 different samples area in high-magnification images (1 µm lateral size, 512 pixels/line resolution).

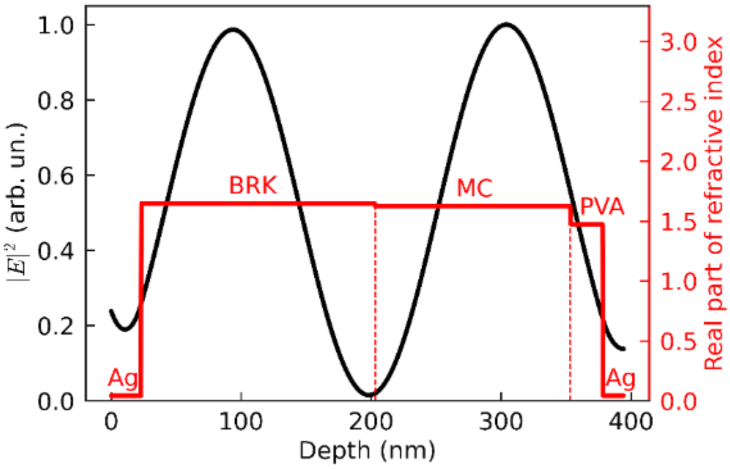

Fig. S2: Electric field distribution ($|E|^2$, black continuous line) and real part of the refractive index (red continuous line) along the cavity sample depth. PVA-BRK and PMMA-MC layers are here indicated as BRK and MC, respectively.

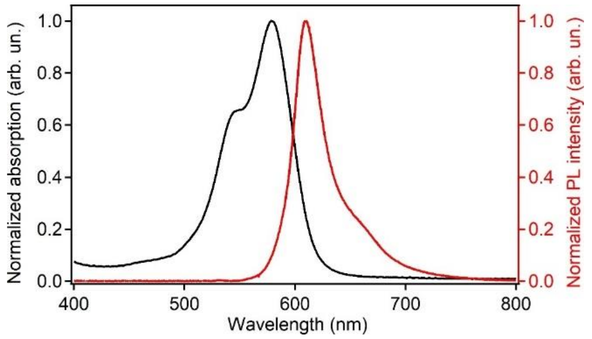

Fig. S3: Absorption and photoluminescence of BRK. Absorption (black continuous line, left vertical scale) and photoluminescence (PL, red continuous line and right vertical scale) spectra of a dilute solution of BRK and PVA in deionized water and methanol mixture (1:1 volumetric ratio). The weight ratio of BRK with respect to PVA is 1:1000, i.e. two orders of magnitude lower than the one used for the thin films with BRK J-aggregates. In such conditions we expect the properties of the BRK molecule to be dominant. The excitation wavelength for the emission measurement is 532 nm. The main peak of the emission spectrum of the dilute solution of BRK in PVA is at about 610 nm. The formation of J-aggregated in thin films obtained by more concentrated solutions red-shifts the absorption spectrum by about 75 nm (see also Fig.1c of the main text).

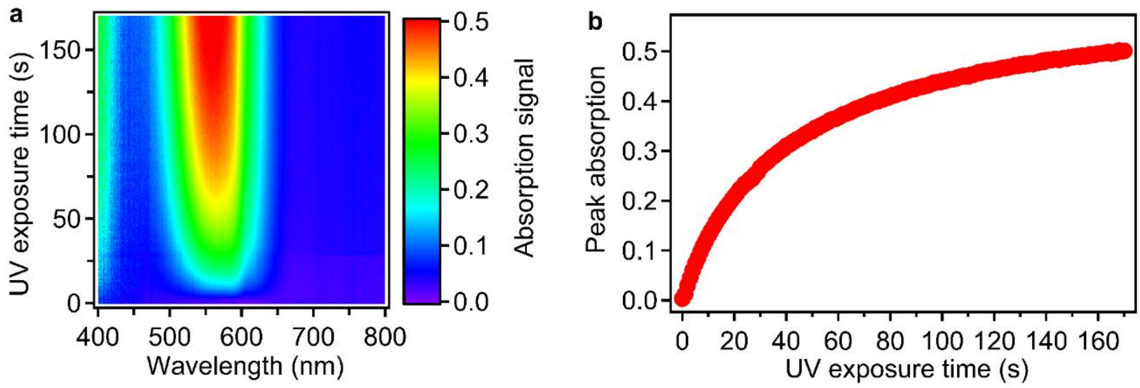

Fig. S4: MC absorption. a Measured absorption spectra of a MC layer upon different UV exposure times. **b** Trend of the absorption peak of the MC at 554 nm (extracted from **a**), *vs*. UV exposure time (365 nm).

2 Sample Fabrication

Three solutions are prepared for spinning the different layers. The first one is made by embedding the spiropyran (SP) molecules in a PMMA matrix. It contains:

- 54 mg of PMMA (Mw: 120k, Sigma-Aldrich)
- 54 mg of SP (TCI)
- 3 mL of toluene (reag. ph. pure ≥ 99.7%, Sigma-Aldrich).

The solution is kept in a sonicator for two hours to dissolve completely the organic materials. The second solution is made by embedding the BRK molecules in a PVA matrix. Materials used are:

- 80 mg of PVA (Mw: 124k-186k, 87-89% hydrolyzed, Sigma-Aldrich)
- 8 mg of BRK (ABCR)
- 2 mL of deionized water
- 2 mL of methanol (Carlo Erba Reagents)

After mechanical mixing, the solution is left for 3 hours on a magnetic stirrer at a temperature of 50°C. The last solution consists of bare PVA, and it is prepared according to the following procedure. Materials used are:

- 50 mg of PVA (Mw: 85k-124k, 99% hydrolyzed, Sigma-Aldrich)
- 4.95 mL of deionized water

After mechanical mixing, the solution is left for 3 hours on the magnetic stirrer at a temperature of 160 °C.

The complete multilayer is obtained by spin coating three films for 60 s on top of each other and on top of a thermally-evaporated Silver mirror (25 nm-thick). Solutions are cast in the following order: PVA solution at 4000 rpm, PMMA-SP solution at 1500 rpm, PVA-BRK solution at 2400 rpm. The cavity is completed by evaporating a top Silver mirror. An example of a sample at three different stages of the process is reported in Fig. S5a-c. A transmission measurement of a Silver mirror evaporated on quartz is shown in Fig. S5d.

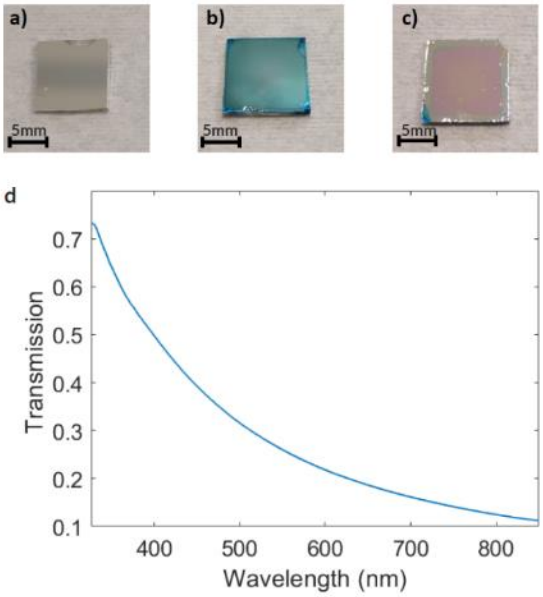

Fig. S5: Microcavity fabrication. Photograph of samples at different stages of fabrication. **a** Silver mirror evaporated on a quartz substrate. **b** Active region embedding both donor and acceptor molecules spin-coated on Silver. **c** Full cavity after the evaporation of 25 nm-thick Silver on top of the active region. **d** Measured optical transmission spectrum of a 25 nm-thick Silver mirror deposited on quartz.

3 Transmission of the microcavity at intermediate times

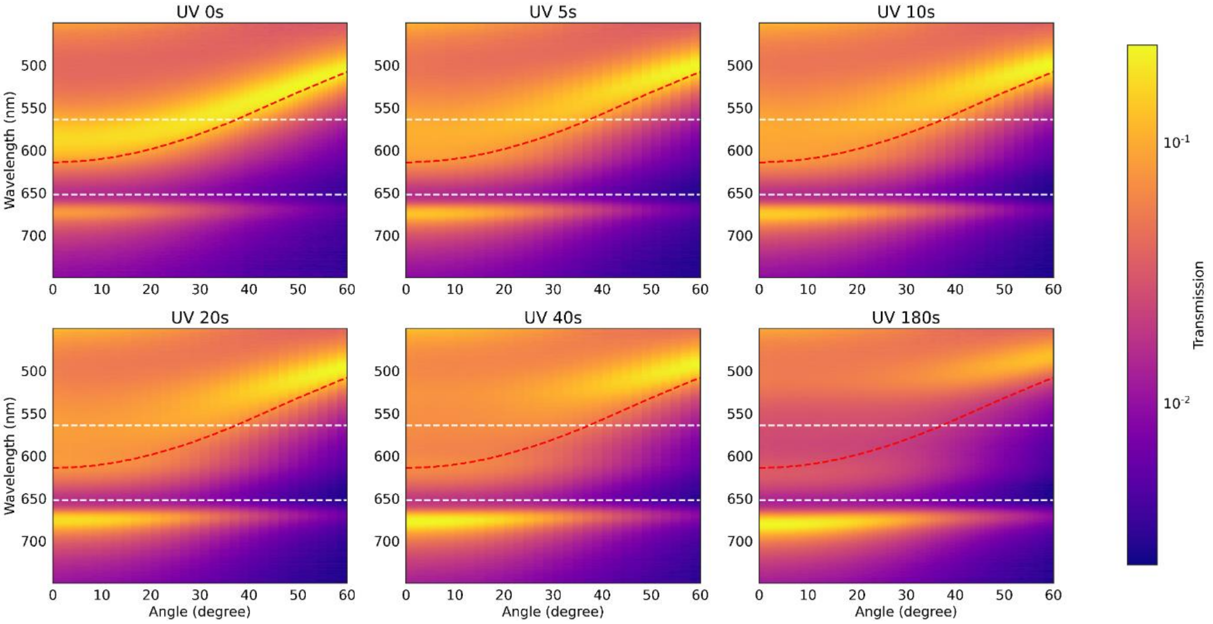

Fig. S6: Cavity transmission measurements. Angle-resolved transmission measurement as a function of UV light exposure time. In the chosen colorscale, unity stands for total transmission. In each colormap the bare cavity mode (red dashed line), the MC excitonic transition (upper white dashed line) and the BRK excitonic transition (lower white dashed line) are also reported.

4 Characterization of BRK and MC first-order cavities

The complex refractive index of the acceptor layer is derived from the characterization of a first-order cavity with PVA-BRK (thickness of the bottom and top Ag mirrors about 25 nm, thickness of the PVA-BRK about 165 nm). The measured and simulated transmission maps are reported in Fig. S7a-b. Using a Transfer Matrix Method (TMM) approach we minimize the difference between the experimental and calculated angle-resolved transmission and reflection spectra. For this procedure we vary as free parameters the layer thicknesses and the parameters for the dielectric permittivity of the active layer. In particular, we approximate the imaginary part of the dielectric permittivity of PVA-BRK as a superposition of 2 Voigt profiles (corresponding to J-aggregates and non-aggregated monomers, respectively). The Voigt profile is a convolution of Gaussian and Lorentzian profiles representing inhomogeneous and homogeneous broadening, respectively. For the dielectric permittivity of BRK we hence use 8 free parameters (2 central frequencies, 2 Gaussian widths, 2 Lorentzian widths, 2 amplitudes). The real part of the permittivity is then calculated by use of Kramers-Kronig relations. As a constant background permittivity, data available in Ref. [1] for the PVA host matrix are used. The obtained refractive index of the PVA-BRK is reported in Fig. S7c-d.

The calculated values are also compared with those obtained from the fit of ellipsometry measurements performed on a PVA-BRK layer spin-coated on a silicon/silicon oxide substrate. The contribution of several oscillators is considered, as indeed visible in the low-wavelength tail of both the *n* and *k* spectra. We find a good agreement between the values of the refractive index obtained by the two different methodologies.

J-aggregates are complex structures and many of their properties strongly depend on the environmental conditions, including the fabrication procedure. The simulations of more complex experiments involving donor and acceptor layers are hence performed by using the refractive index values of PVA-BRK calculated by the TMM fitting, which comes from materials undergone the same cavity fabrication processes.

A characterization of a first-order cavity with only PMMA-SP/MC is also performed. The active region consists of two 25 nm thick PVA buffer layers (in contact with the 25 nm thick Ag mirrors) and a central layer of PMMA-SP/MC matrix with a thickness about 110 nm. The experimental and simulated transmission maps are reported in Fig. S8a-b. Analogously to the procedure described above for BRK, we approximate the dielectric permittivity of PMMA-MC as a superposition of two Voigt profiles, with a constant background permittivity due to the PMMA host matrix taken from Ref. [2]. Then, we fit angle-resolved spectra using the TMM and, through a best fit procedure, we retrieve the optimal parameters that allow us to get the refractive index of the PMMA-MC (Fig. S8c-d). The permittivity of PMMA-SP in the relevant spectral range is simulated as the permittivity of PMMA with a small constant absorption. The latter value is obtained through fitting of the corresponding transmission spectra of the cavity with SP layer. The curve obtained from the fit of the refractive index of the PMMA-SP and PMMA-MC is further exploited for simulations of more complex experiments involving donor and acceptor layers, as described in Methods of the main manuscript.

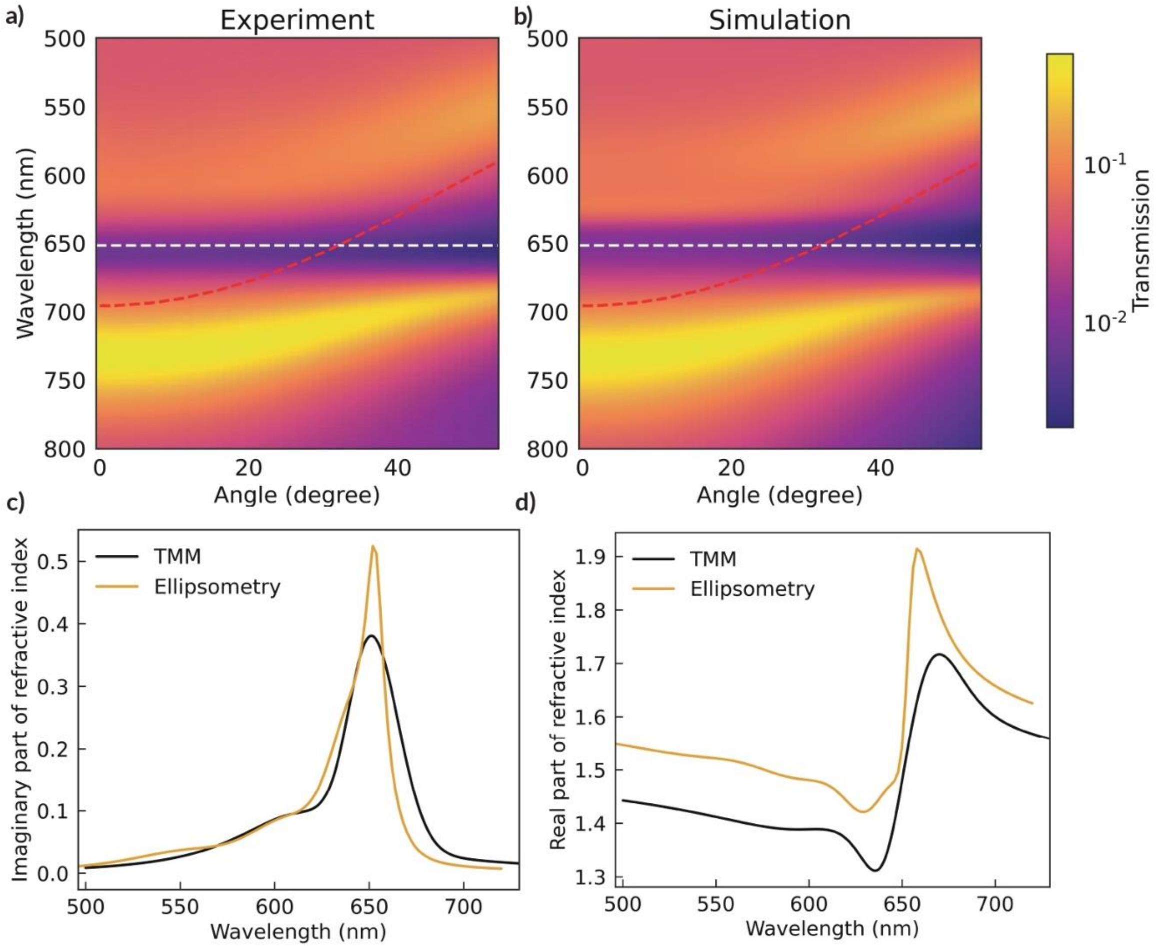

Fig. S7: BRK cavity transmission and PVA-BRK refractive index. Experimental **a** and simulated **b** angle-resolved transmission maps of a first-order cavity embedding a PVA-BRK layer. Imaginary **c** and real **d** part of the BRK layer refractive index, as calculated by minimizing the difference between the experimental and calculated angle-resolved transmission and reflection spectra of the first-order cavity (TMM, black line) and by ellipsometry data (yellow line).

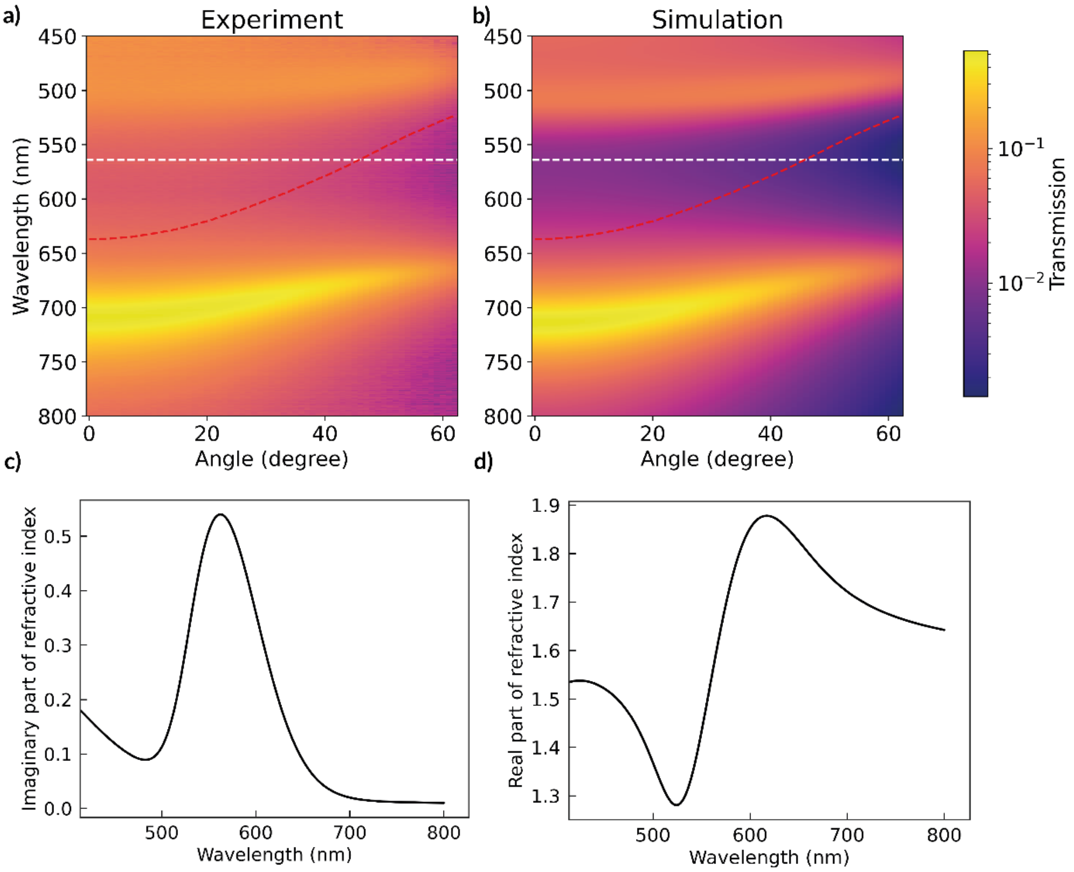

Fig. S8: MC cavity transmission and PMMA-MC refractive index. Experimental **a** and simulated **b** angle-resolved transmission maps of a first-order cavity embedding only the PMMA-MC layer. Fitted imaginary **c** and real **d** part of the PMMA-MC layer refractive index.

5 Coupled Oscillators Model

Each polaritonic dispersion is fitted with a coupled oscillators model for each UV exposure time. The method consists in solution of the eigenvalue problem:

$$\begin{bmatrix} \omega_{\mathrm{BRK}} & 0 & g_{\mathrm{BRK}} \\ 0 & \omega_{\mathrm{MC}} & g_{\mathrm{MC}}(t_{\mathrm{exp}}) \\ g_{\mathrm{BRK}} & g_{\mathrm{MC}}(t_{\mathrm{exp}}) & \omega_{\mathrm{cav}}(\theta) \end{bmatrix} \begin{bmatrix} \alpha_{\mathrm{BRK}}(\theta, t_{\mathrm{exp}}) \\ \alpha_{\mathrm{MC}}(\theta, t_{\mathrm{exp}}) \\ \alpha_{\mathrm{cavity}}(\theta, t_{\mathrm{exp}}) \end{bmatrix} = \omega_{\mathrm{pol}}(\theta, t_{\mathrm{exp}}) \begin{bmatrix} \alpha_{\mathrm{BRK}}(\theta, t_{\mathrm{exp}}) \\ \alpha_{\mathrm{MC}}(\theta, t_{\mathrm{exp}}) \\ \alpha_{\mathrm{cavity}}(\theta, t_{\mathrm{exp}}) \end{bmatrix} \quad \text{(S1)}$$

where ω_{BRK}, ω_{MC} and ω_{cav} are frequencies of the BRK transition, the MC transition and the cavity mode, respectively; g_{BRK} and $g_{MC}(t_{\mathrm{exp}})$ are coupling strengths between the emitters and the cavity mode; θ is the measurement angle. The eigenfrequencies $\omega_{pol}(\theta, t_{exp})$ represent the frequencies of hybrid polaritonic states, and eigenvectors carry information about Hopfield coefficients which are equal to: $|\alpha_i|^2$ (i=BRK, MC, cavity). The peak positions of the emitter absorption spectra are taken as their transition frequencies. The frequency of the bare cavity mode is retrieved from TMM simulations. The estimated Hopfield coefficients for each polaritonic branch for three different exposure times are shown in Figures S9, S10, S11.

In order to get coupling strengths corresponding to the experiment we perform one fitting procedure for all the exposure times t_{exp}, where we vary g_{BRK} and g_{MC} trying to minimize the difference between $\omega_{pol}(\theta, t_{exp})$ and the spectral position of the polaritonic states taken from experimental angle-resolved transmission spectra. It should be noted that for every exposure time we take different coupling strength for the donor (since we expected an increase of the number of MC molecules) and kept fixed the coupling strength between acceptor molecules and cavity mode. The dependence of $g_{MC}(t_{exp})$ is reported in the Fig. S12.

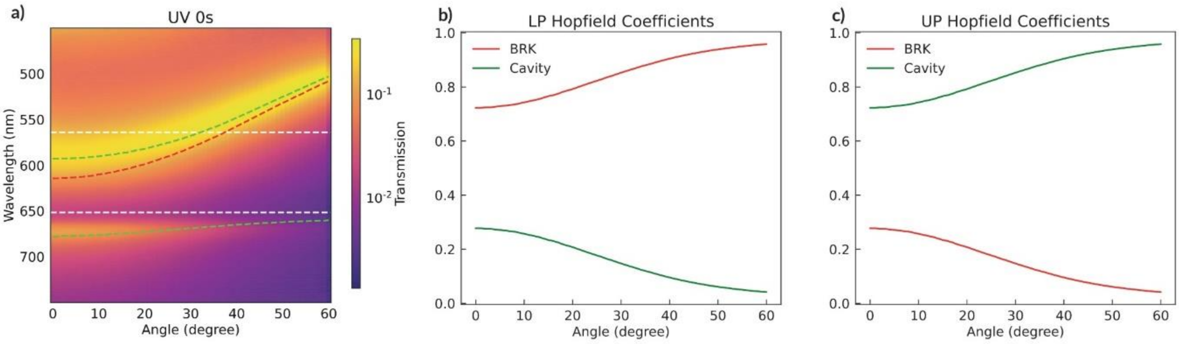

Fig. S9: Pristine cavity transmission and Hopfield coefficients. a Colormap of the microcavity transmission at 0 s of UV exposure and relative angular dispersion of the Hopfield coefficients for the LP **b** and UP **c** branches. The colorscale is chosen to be logarithmic. In the colormap the bare cavity mode (red dashed line), the MC excitonic transition (upper white dashed line) and the BRK excitonic transition (lower white dashed line) are also reported. The green dashed lines are the result of a fit using the coupled oscillators model.

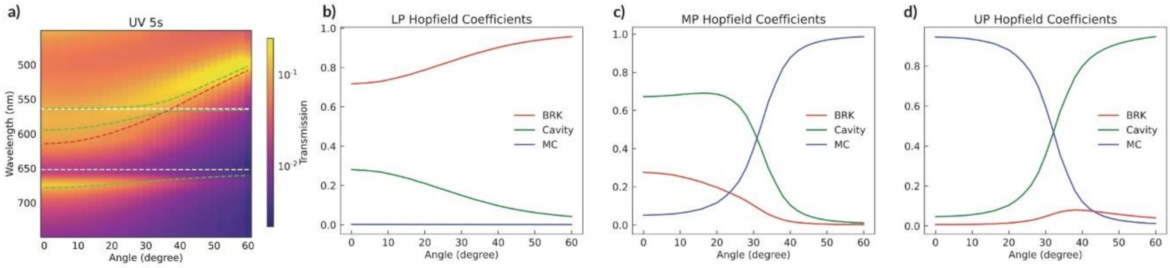

Fig. S10: Cavity transmission at 5 s and Hopfield coefficients. a Colormap of the microcavity transmission at 5 s of UV exposure and relative angular dispersion of the Hopfield coefficients for the LP **b**, MP **c** and UP **d** branches. In the colormap the bare cavity mode (red dashed line), the MC excitonic transition (upper white dashed line) and the BRK excitonic transition (lower white dashed line) are also reported. The green dashed lines are the result of a fit using the coupled oscillators model.

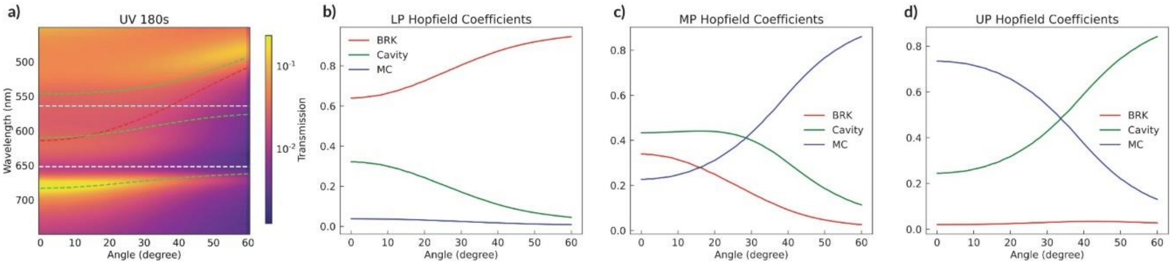

Fig. S11: Cavity transmission at 180 s and Hopfield coefficients. a Colormap of the microcavity transmission at 180 s of UV exposure and relative angular dispersion of the Hopfield coefficients for the LP **b**, MP **c** and UP **d** branches. In the colormap the bare cavity mode (red dashed line), the MC excitonic transition (upper white dashed line) and the BRK excitonic transition (lower white dashed line) are also reported. The green dashed lines are the result of a fit using the coupled oscillators model.

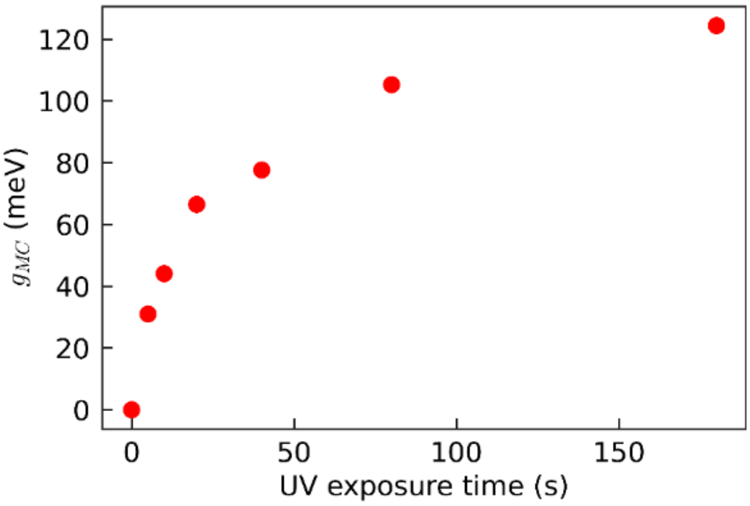

Fig. S12: MC coupling coefficient. Collective light-matter coupling strength of the MC molecules as a function of the UV exposure time.

6 Analysis of the MC to SP back-conversion

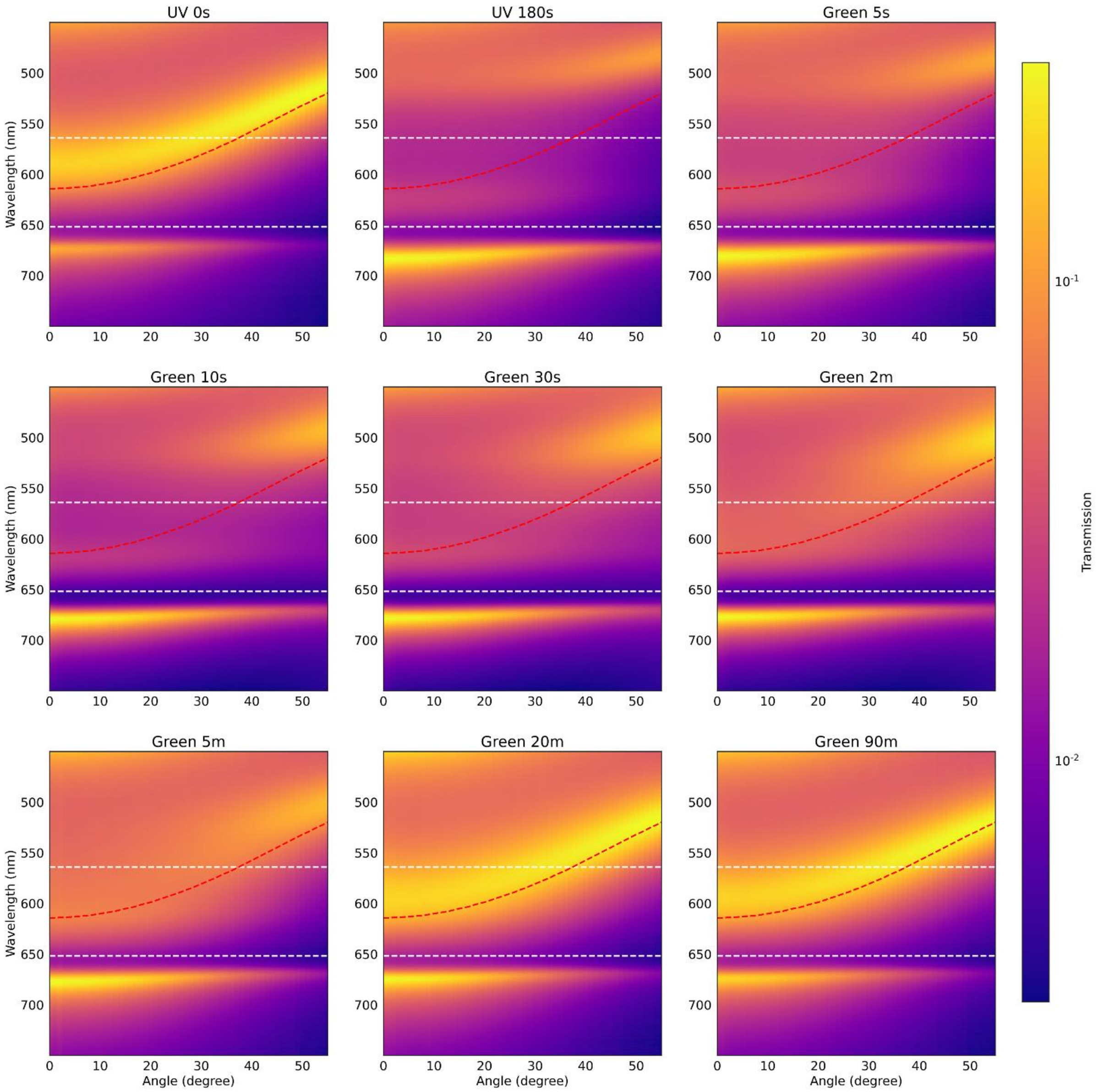

Fig. S13: Cavity transmission during MC back-switching. Angle-resolved transmission spectra as a function of UV and green light exposure time. In each colormap the bare cavity mode (red dashed line), the MC excitonic transition (upper white dashed line) and the BRK excitonic transition (lower white dashed line) are also reported.

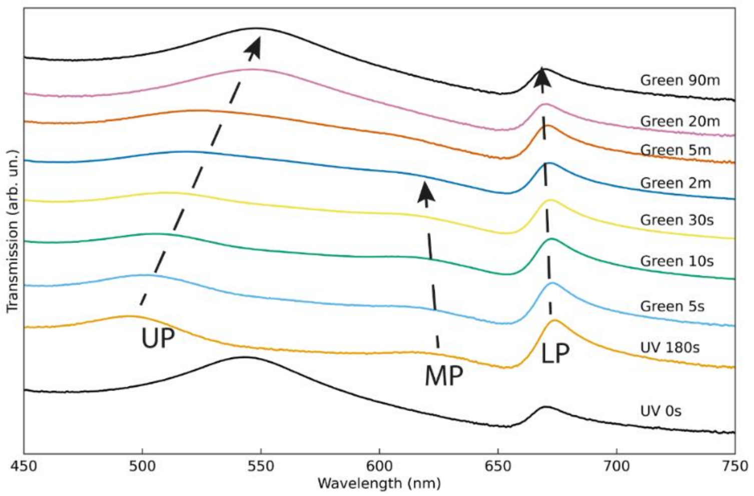

Fig. S14: Cavity transmission spectra at fixed angle. Transmission spectra measured at 39° incidence angle as a function of UV and green light exposure times. The black arrows highlight the shift of the UP, MP and LP branches, respectively. The spectra are vertically shifted for better clarity.

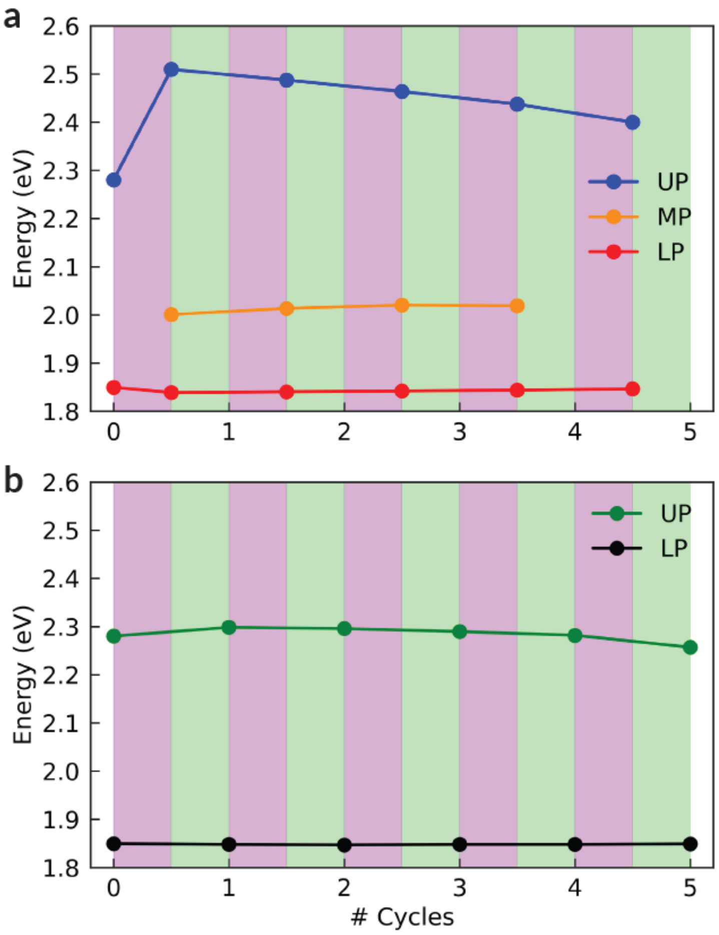

Fig. S15: Polariton behavior upon UV/green irradiation cycles. Energy of the UP, MP and LP branches after repeated UV/green irradiation. The energy values are obtained from transmission spectra collected at 39° after each UV (**a**) or green (**b**) exposure step.

7 PL of the microcavity at intermediate UV exposure times

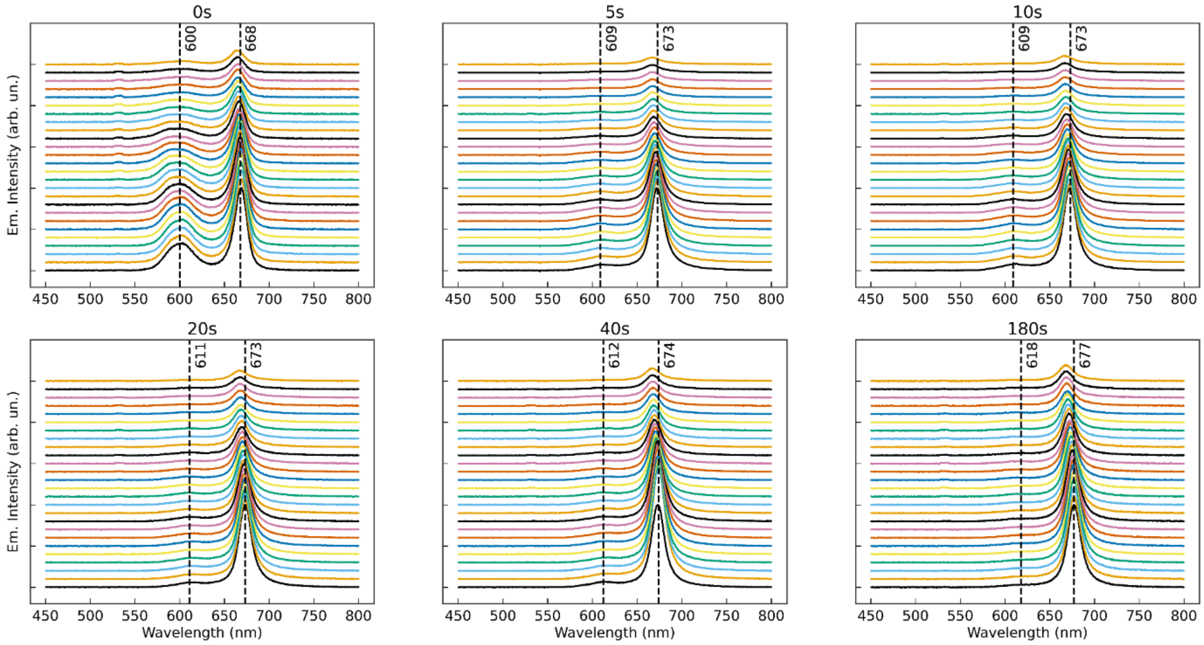

Fig. S16: Cavity emission during SP to MC conversion. Experimental angle-resolved emission spectra of the cavity as a function of the initial UV light exposure time. The excitation wavelength for the emission measurement is 532 nm. Each spectrum is measured every 2° starting from the bottom of the plot. For each UV exposure time, spectra are normalised to the maximum emission intensity of the one measured at 0°. The vertical dashed lines identify the peaks wavelength (nm) at 0° of emission.

8 Analysis of the cavity PL upon MC-to-SP back-switching

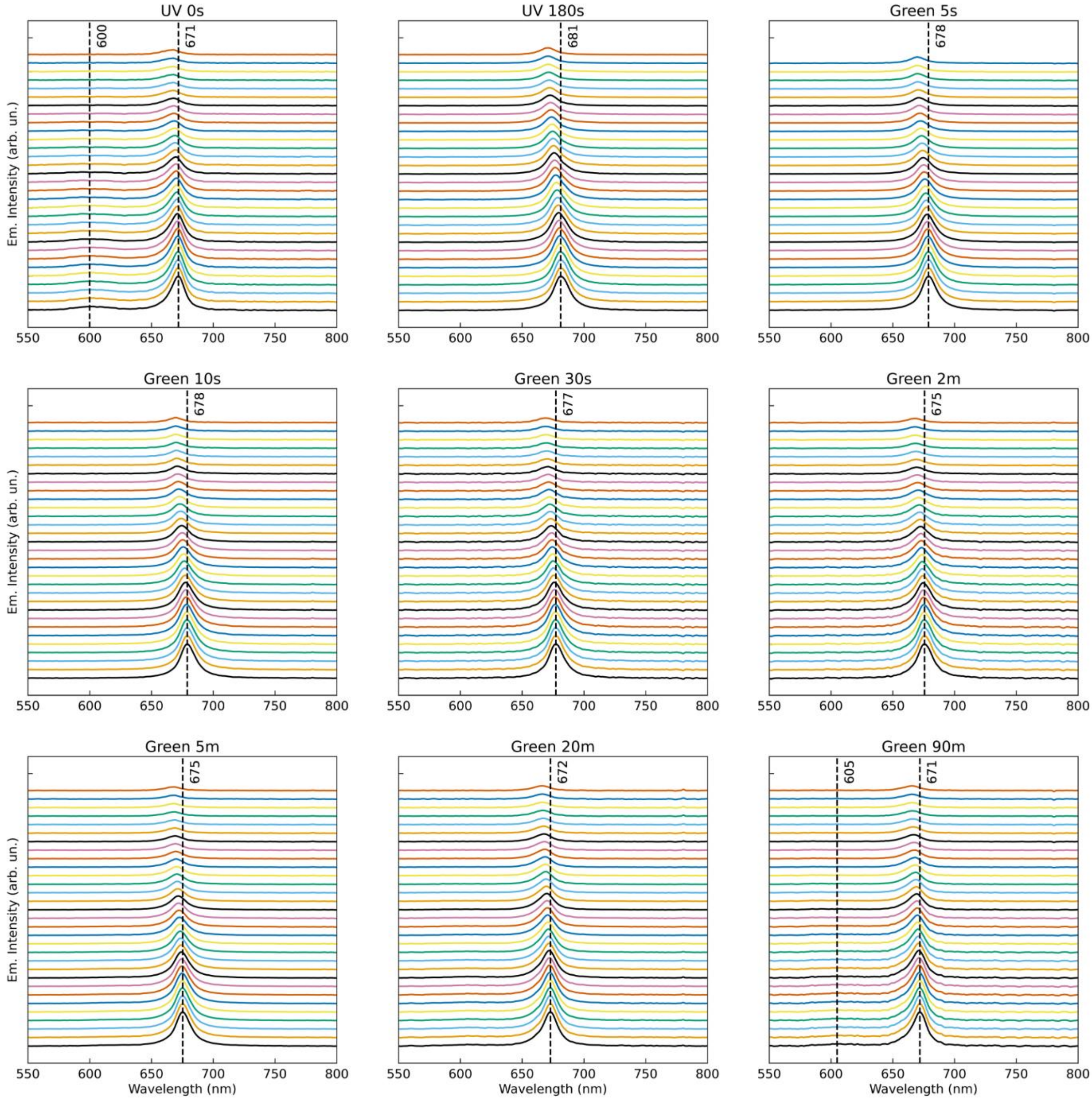

Fig. S17: Cavity emission during MC back-switching. Experimental angle-resolved PL spectra of the cavity as a function of the initial UV and subsequent green light exposure times. Excitation wavelength: 532 nm. Spectra are vertically shifted. Each spectrum is measured at a given angle value, each with 2° spacing from the previous one, starting from the bottom of the plot. The spectra are all normalised to the maximum emission intensity of the 0° spectrum. The dashed vertical lines identify the peaks wavelength at 0°.

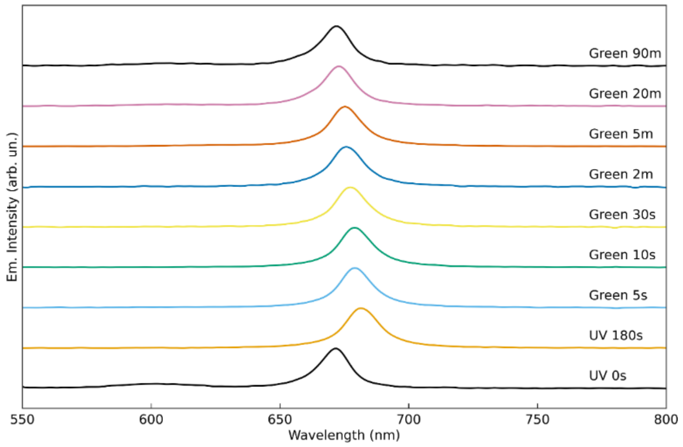

Fig. S18: Cavity emission during MC back-switching at fixed angle. PL spectra of the cavity as a function of the initial UV and subsequent green light exposure times measured at 0°. Excitation wavelength for the emission measurements: 532 nm. Each spectrum is normalized to its maximum intensity value. The spectra are vertically shifted for better clarity.

9 The model for the cavity emission

We assume that the cavity emission signal can be regarded as emission of each molecular species modulated by an effective filtering induced by the cavity. Thus, we use the following expression:

$$I_{cav}(\omega,\theta;t_{exp}) = \alpha(t_{exp})\, F_{BRK}(\omega,\theta;t_{exp}) I_{BRK}(\omega;t_{exp}) + \beta(t_{exp}) F_{SP/MC}(\omega,\theta;t_{exp}) I_{SP/MC}(\omega;t_{exp}) \qquad \text{(S2)}$$

where F_i(i=BRK, SP/MC) is the filter function for the emission of molecular layer i. Since the emission of each active layer goes from the middle of the sample to the detector located behind one of the mirrors, for a correct estimation of F_i, the corresponding transmission coefficient should be considered. So F_i can be expressed as:

$$F_i(\omega,\theta;t_{exp}) \propto \int_{\substack{i-th \\ layer}} T_{x\to det}(x;\omega,\theta,t_{exp})\, dx \qquad \text{(S3)}$$

where $T_{x\to det}$ represents the transmission of the light from the plane x of the layer i to the detector. This quantity can be easily calculated by TMM. Since $T_{x\to det}$ strongly depends on the position of the source x, we (incoherently) integrate the emission contributions coming from the different parts of the layer i. The results obtained with the expression for F_i are presented in Fig. S19.

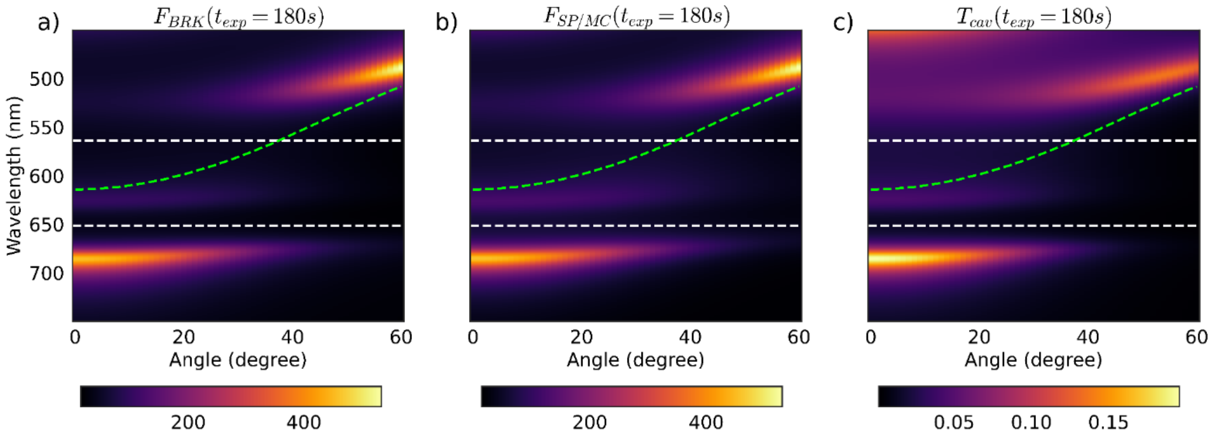

Fig. S19: a,b Filters for molecular emission at 180 s of UV exposure, for BRK and MC layers, respectively. **c** Simulated transmission of the cavity at t_{exp} = 180 s. In each colormap the bare cavity mode (green dashed line), the MC excitonic transition (upper white dashed line) and the BRK excitonic transition (lower white dashed line) are also reported.

It can be seen that for both molecular species, the maps are similar to the total cavity transmission. The main deviations are in the short-wavelength region, which is off-resonant from the emission of the molecular species. We also note that the scale of BRK and SP/MC filter functions are similar, so that the cavity transmission is a good approximation for the emission filter for both molecular species. Additionally, the difference in absolute scale between the filter functions and cavity transmission does not play any role, since for the analysis we use normalized weight coefficients.

10 Rate equations model

In order to validate the approach that we use to describe emission dynamics for our system, we also implement a rate equations model for the lower polariton branch (LPB)[3]. Since the photochemical reaction has a rate comparatively small to the polariton dynamics we can a use steady state condition for the population of the LPB:

$$\frac{dN_{\mathrm{LPB}}}{dt} = 0 = C_1 \left|\alpha_A^{LPB}\right|^2 + C_2 \left|\alpha_D^{LPB}\right|^2 + C_3 P_{\mathrm{LPB}} \left|\alpha_{\mathrm{ph}}^{LPB}\right|^2 - C_4 N_{\mathrm{LPB}} \left|\alpha_{\mathrm{ph}}^{LPB}\right|^2 - C_5 N_{\mathrm{LPB}} \left|\alpha_A^{LPB}\right|^2 - C_6 N_{\mathrm{LPB}} \left|\alpha_D^{LPB}\right|^2 \quad \text{(S4)}$$

where N_{LPB} is the lower polariton branch population, $\left|\alpha_i^{LPB}\right|^2$ (i=A, D, ph) is the LPB Hopfield

coefficient for acceptor, donor and cavity photon respectively, and P_{LPB} is the overlap between LPB and emission spectra of the bare molecules. In this equation, the first two terms describe the processes of vibrational scattering from donor and acceptor excitonic reservoirs, the third term denotes the radiative pumping, the fourth one stands for radiative decay through the photonic component of lower polariton, and the last two terms describe relaxation to excitonic reservoirs.

We assume that for our system radiative pumping dominates over vibrational scattering, since the LPB significantly overlaps with the emission spectra of both molecular species. Also, we disregard the last term in the equation, assuming that its contribution to the LPB population is negligible compared to the radiative mechanism and LP relaxation to the acceptor reservoir, in full accordance with previous studies[3-5].

$$N_{LPB} = \frac{C_1|\alpha_A^{LPB}|^2 + C_2|\alpha_D^{LPB}|^2 + C_3 P_{LPB}\left|\alpha_{ph}^{LPB}\right|^2}{C_4\left|\alpha_{ph}^{LPB}\right|^2 + C_5|\alpha_A^{LPB}|^2 + C_6|\alpha_D^{LPB}|^2} \approx \frac{C_3 P_{LPB}\left|\alpha_{ph}^{LPB}\right|^2}{C_4\left|\alpha_{ph}^{LPB}\right|^2 + C_5|\alpha_A^{LPB}|^2} \qquad \text{(S5)}$$

Since the emission intensity $I_{LPB} \propto N_{LPB}|\alpha_{ph}^{LPB}|^2$, we can write:

$$I_{LPB} \propto \frac{P_{LPB}\left|\alpha_{ph}^{LPB}\right|^2}{1+R}, \qquad \text{(S6)}$$

where $R = \frac{C_5\left|\alpha_A^{LPB}\right|^2}{C_4\left|\alpha_{ph}^{LPB}\right|^2}$ is the ratio between the rates defining LP relaxation into acceptor reservoir and LP radiative decay, respectively. This ratio can be estimated using TMM. In order to obtain R we performed simulations calculating the power emitted into free space and absorbed in the acceptor molecules for emission from the donor molecules. In this scenario, we can estimate the rate of LP decay as the power emitted when the sources are distributed over the donor layer (P_{out}). LP relaxation to the acceptor reservoir then corresponds to the absorption of light emitted from the donor layer (A_{acc}). The latter process is mediated by radiative modes of the hybrid system (polaritons) and, thus, corresponds to the cascaded mechanism we aim to describe (donor → LP,MP,UP → acceptor). However, since only the LP is close to resonant

with the acceptor reservoir, the acceptor absorption is mostly mediated by the LP. The resulting frequency-dependent rates for the case of 0 degree, 180s of UV illumination are presented in Fig. S20. The integrated ratio $R = 1.19$ for this case, which means that in our system LP relaxation to the acceptor reservoir is of the same order as LP radiative decay.

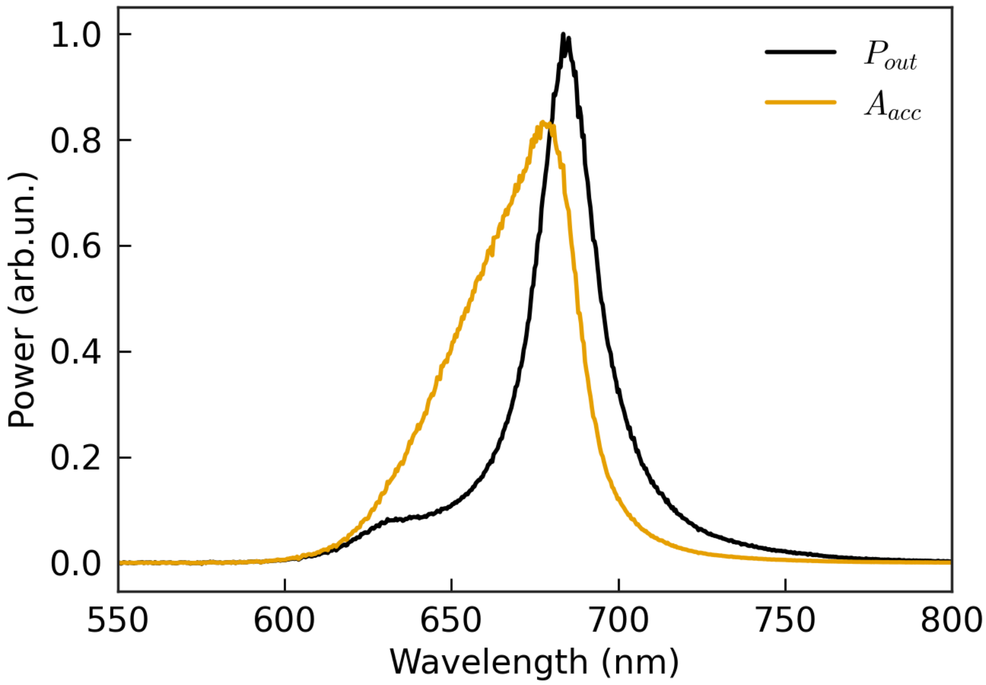

Fig. S20: Power leaving the cavity due to donor emission (black), acceptor absorption following the donor emission (yellow) at 0 degree, 180s of UV illumination.

The equation S6 is equivalent to the approach we use for analysis of the emission properties of the hybrid system. The comparison of the two approaches with experimental data are reported in Fig. S21. In order to calculate the radiative pumping contribution for the rate equation approach we retrieve the LPB from the cavity transmission spectra. Since for high angles this peak is barely visible, we compute the graphs only for angles smaller than 30°. It could be noticed that the approach we used for the emission dynamics analysis is able to reproduce the same behavior as experimental data and gives qualitatively the same results as the rate equations method, which, in turn, proves the validity of aforementioned assumptions.

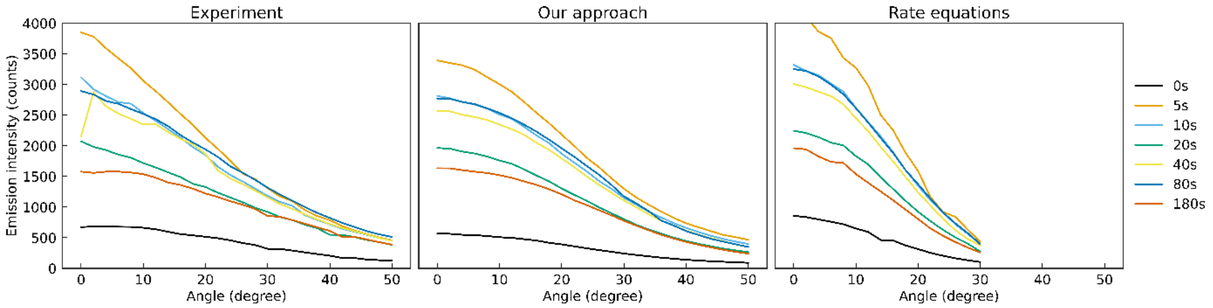

Fig. S21: Emission intensity from lower polaritonic state for different times from the start of reaction. Left plot: experimental data; middle plot: results obtained using our approach described in the main text; right plot: the results for rate equations approach.

11 PL properties of the molecules outside and inside the cavity

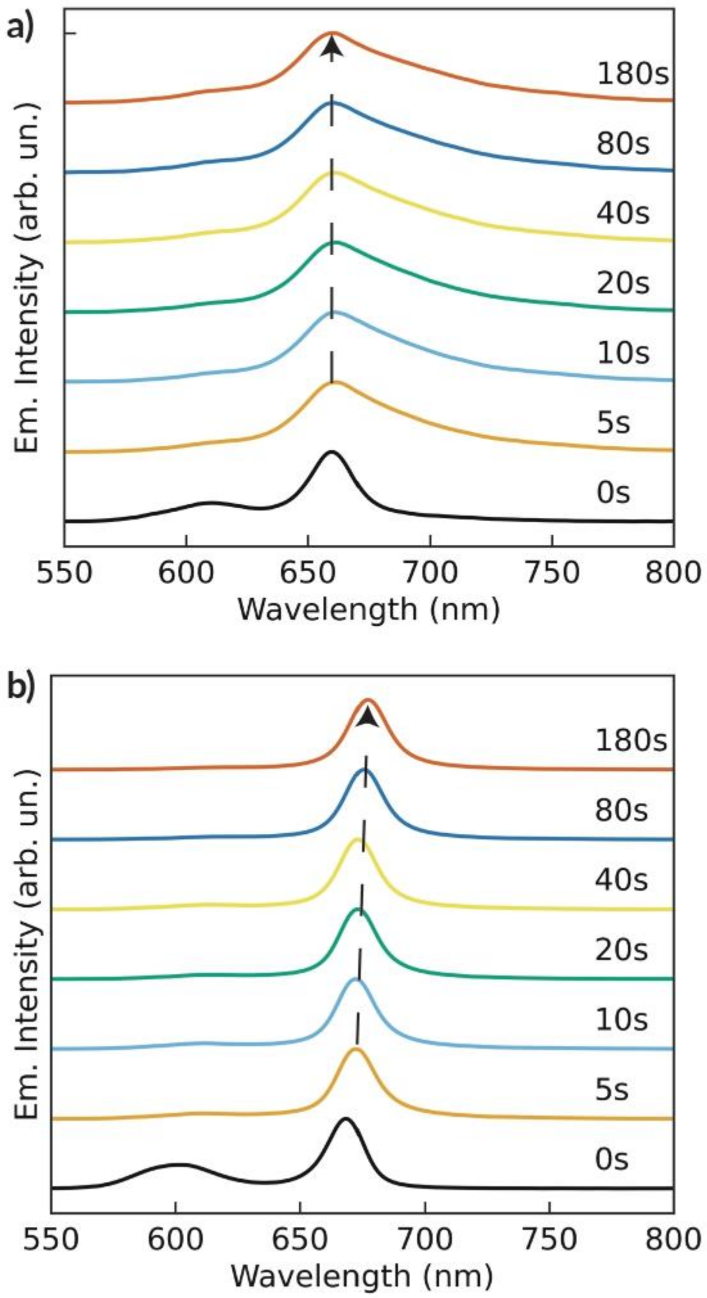

Fig. S22: Emission spectra of the photo-active multilayer with BRK and MC molecules out of cavity (**a**) and in the cavity (**b**), upon varying the UV exposure times between 0 s and 180 s. A green laser at 532 nm is used for PL excitation. Spectra are vertically shifted, and each spectrum is normalised to its maximum intensity value.

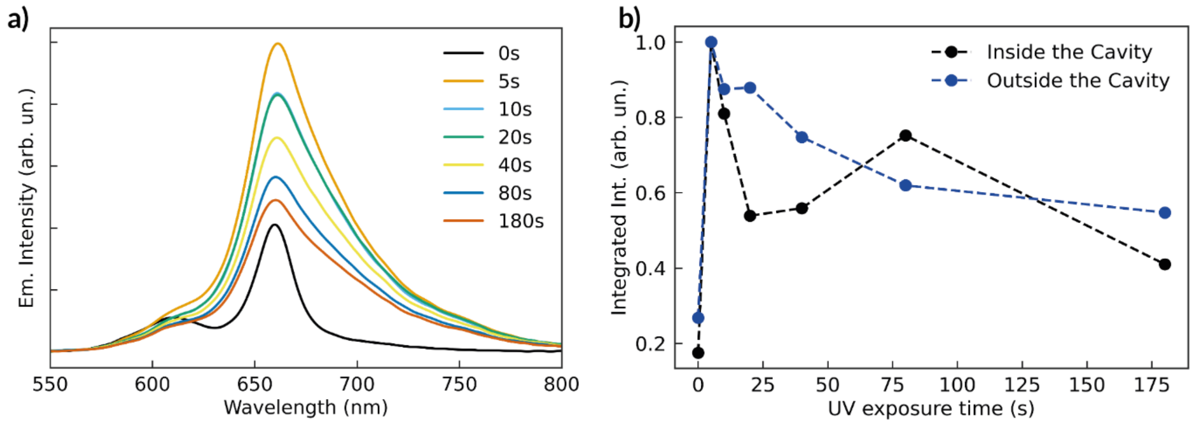

Fig. S23: Emission properties of the photo-active multilayer with BRK and MC molecules. **a** Emission spectra of the multilayer outside the cavity acquired at 0° as a function of the UV light exposure time, by using a green laser at 532 nm for PL excitation. **b** Comparison of the integrated emission intensity of the multilayer inside the cavity and outside the cavity (at 0°).

12 Cavity with only the acceptor

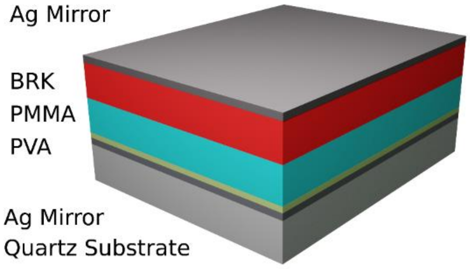

Fig. S24: Schematics of the realized acceptor-only cavity.

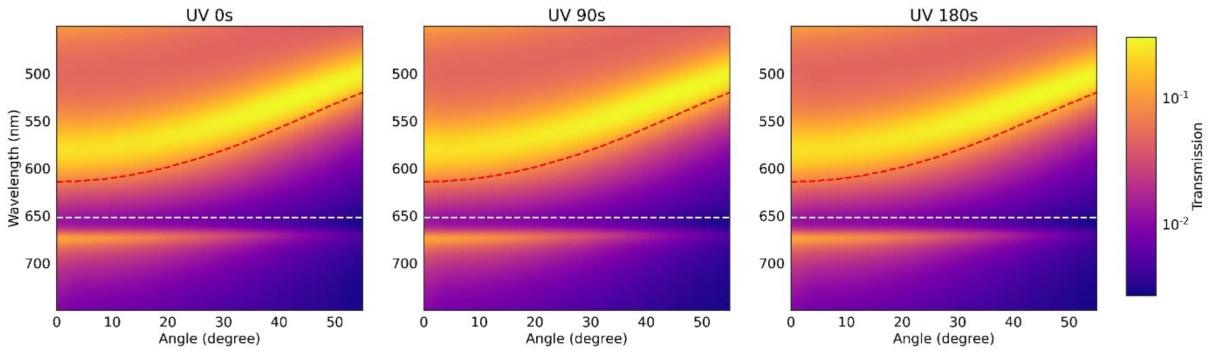

Fig. S25: Acceptor-only cavity transmission measurements. Angle-resolved transmission measurement as a function of UV light exposure time. In each colormap the bare cavity mode (red dashed line) and the BRK excitonic transition (white dashed line) are reported.

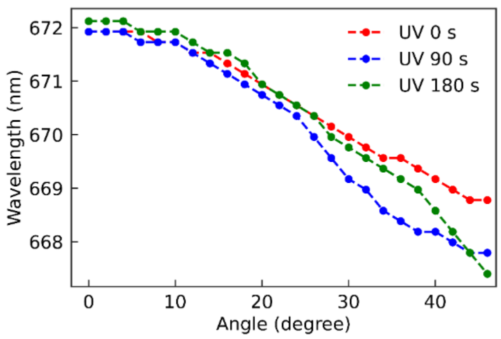

Fig. S26: Acceptor-only cavity emission measurements. Angular dependence of the wavelength of the cavity emission peak at different UV exposure times. The excitation wavelength for the emission measurement is 532 nm.

13 Cavity with only the donor

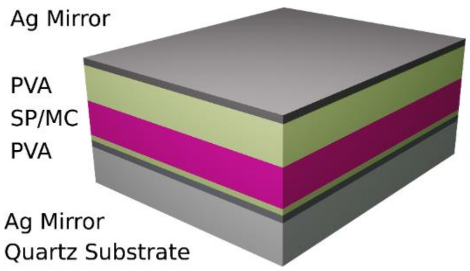

Fig. S27: Schematics of the realized donor-only cavity.

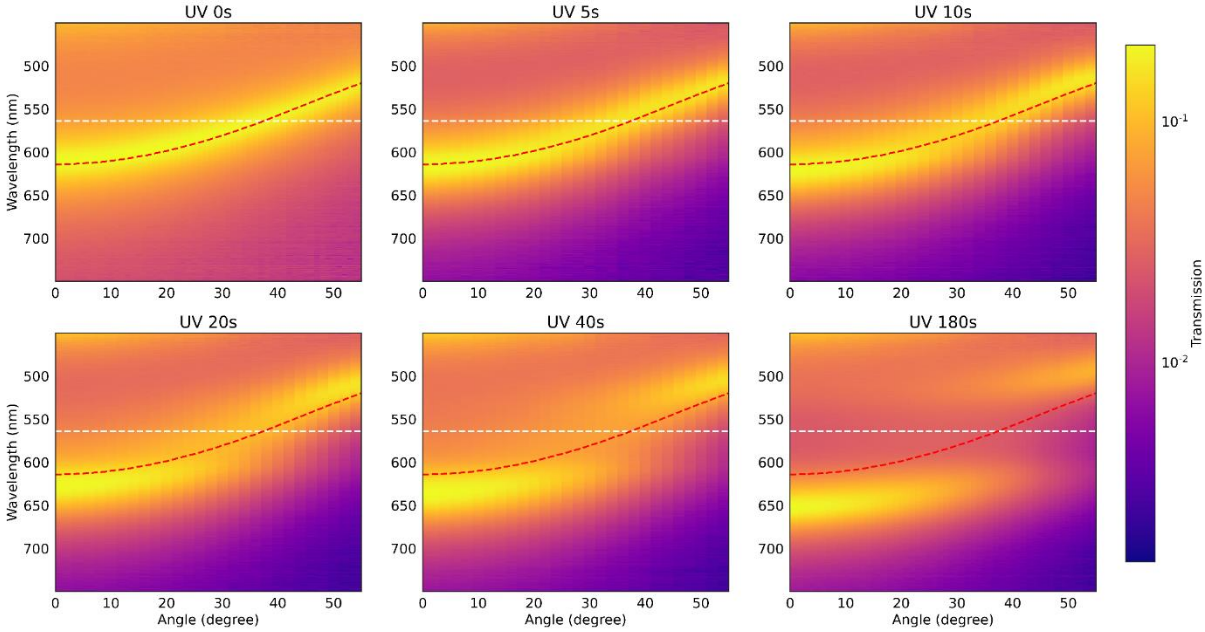

Fig. S28: Donor-only cavity transmission measurements. Angle-resolved transmission measurement as a function of UV light exposure time. In each colormap the bare cavity mode (red dashed line) and the MC excitonic transition (white dashed line) are reported.

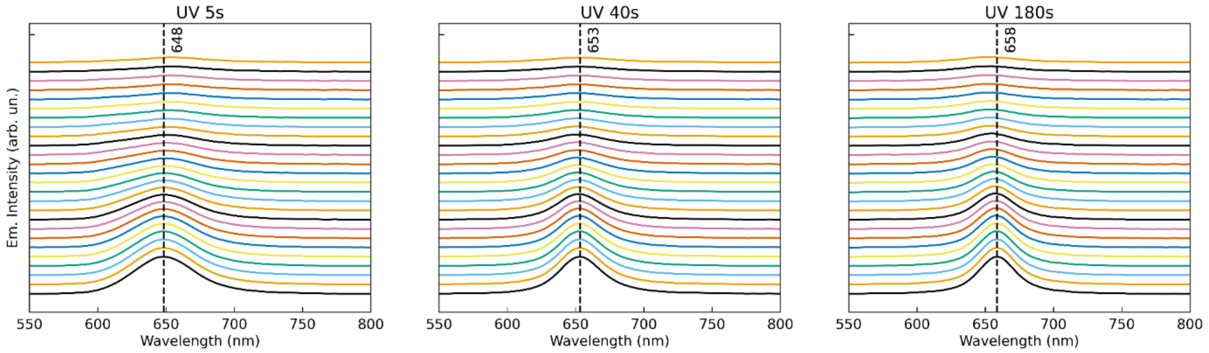

Fig. S29: Donor-only cavity emission measurements. Experimental angle-resolved emission spectra of the donor-only cavity as a function of the UV light exposure time. The excitation wavelength for the emission measurement is 532 nm. For each UV exposure time, spectra are normalised to the maximum emission intensity of the one measured at 0°. The vertical dashed lines identify the peaks wavelength (nm) at 0°.

14 Calculation of the BRK and MC contributions to the emission pattern of the multilayer outside the cavity

In order to find the donor and acceptor contributions to the emission, we approximate the PL signal of the multilayer by the following formula:

$$\boldsymbol{I_{ML}(\omega; t_{exp}) = \alpha_{ML}(t_{exp}) I_{BRK}(\omega) + \beta_{ML}(t_{exp}) I_{SP/MC}(\omega; t_{exp})} \quad \text{(S7)}$$

where $\boldsymbol{I_{BRK}}$, $\boldsymbol{I_{SP/MC}}$ are the emission intensities of the molecules outside the cavity, and $\boldsymbol{\alpha_{ML}}$, $\boldsymbol{\beta_{ML}}$ are phenomenological weight coefficients for BRK and MC, respectively. The coefficients and $\boldsymbol{\alpha_{ML}}$, $\boldsymbol{\beta_{ML}}$ are obtained by fitting the time-dependent spectra $\boldsymbol{I_{ML}(\omega; t_{exp})}$ to the experimental spectra shown at the Fig. S23a using the weight coefficients $\boldsymbol{\alpha_{ML}}$ and $\boldsymbol{\beta_{ML}}$ as free parameters. Some examples of the results of the fitting are shown in Fig. S30.

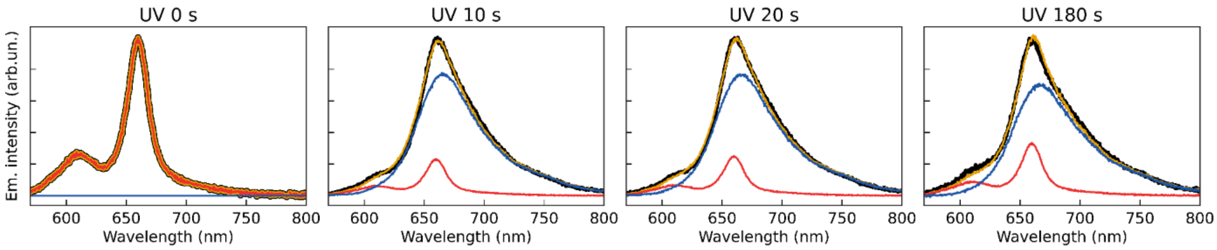

Fig. S30: Fitting of the multilayer emission spectra outside the cavity. Experimental (black) and simulated (yellow) emission spectra for multilayer outside the cavity for exposure time of 0 s, 10 s, 20 s, 180 s using a laser at 532 nm as a pump. BRK contribution $\alpha_{ML}(t_{exp}) I_{BRK}(\omega)$ (red) and SP/MC contribution $\beta_{ML}(t_{exp}) I_{SP/MC}(\omega; t_{exp})$ (blue) into the emission pattern.

As can be seen from Fig. S30, the emission spectra of the multilayer outside the cavity can be very well described as linear superposition of the spectra of separate molecular species. The temporal dynamics of the normalized weight coefficients $\alpha_n = \frac{\alpha_{ML}}{\alpha_{ML}+\beta_{ML}}$ and $\beta_n = \frac{\beta_{ML}}{\alpha_{ML}+\beta_{ML}}$ is presented in Fig. 6b of the main text.

15 Model sensitivity to experimental parameter variations

The intensity of the effective excitation can vary during the experiments. This variation may be related to the excitation laser or to variations in the excitation efficiency within the cavity. The latter is affected by various factors such as changes in refractive index due to UV exposure and modifications in the cavity transparency resulting from shifts in polaritonic states.

The changes of effective pumping can significantly affect the overall emission from the sample, which in turn leads to variations in the weight coefficients α and β used for the emission analysis. However, both α and β should be proportional to the excitation intensity I_{exc}, as the absorbance of molecules and, in turn, the emission intensity both depend linearly on I_{exc}. The subsequent normalization of the weight coefficients that we perform has the result that $\alpha_n = \frac{\alpha}{\alpha+\beta}$ and $\beta_n = \frac{\beta}{\alpha+\beta}$ do not depend on the excitation intensity. Normalizing the weight coefficients also allows for a fair comparison of results obtained inside and outside the cavity, provided that the thickness ratio of the active layers remains constant.

It is also important to note that emission of the multilayer structure, both inside and outside the cavity, is affected by the emission pattern of the SP/MC layer in PMMA. This pattern is significantly modified throughout the UV exposure due, on one side, to the increase of MC concentration and, on the other side, to the concomitant photo-bleaching effects related to photo-oxidation and other fatigue or aggregation mechanisms impacting on the merocyanine molecules[6-9], as evidenced by the emission spectra $I_{SP/MC}(\omega; t_{exp})$ (Fig. S23a). However, this does not affect the weight coefficients since these effects are already accounted for in Eq. 1 of

the main text and in Eq. S7 of SI, which consider the quantity $I_{SP/MC}(\omega; t_{exp})$.

16 Dependence of energy transfer on cavity detuning

To investigate the underlying nature of the observed results, we performed analogous experiments with the cavity intentionally off-resonant to the molecular species. To achieve this, we realized a cavity with BRK and SP/MC layer thicknesses 240 nm and 225 nm, respectively. It is worth noting that in the resonant cavity the thicknesses are 180 nm and 150 nm. Hence the choice of layer thickness values ensures that the ratio between them remained approximately constant for a meaningful comparison. The distribution of the electric field inside the off-resonant cavity is show in Figure S31.

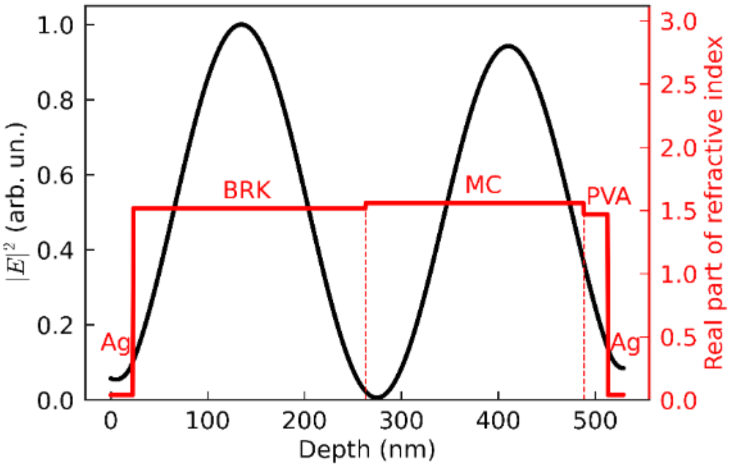

Fig. S31: Electric field distribution ($|E|^2$, black continuous line) and real part of the refractive index (red continuous line) along the cavity sample depth for the off-resonant cavity. PVA-BRK and PMMA-MC layers are here indicated as BRK and MC respectively.

In Figure S32, we present the transmission of the off-resonant cavity sample at 0-180 s UV exposure times. Additionally, in the Figure S33 we display the angle-resolved emission spectra of the same cavity sample.

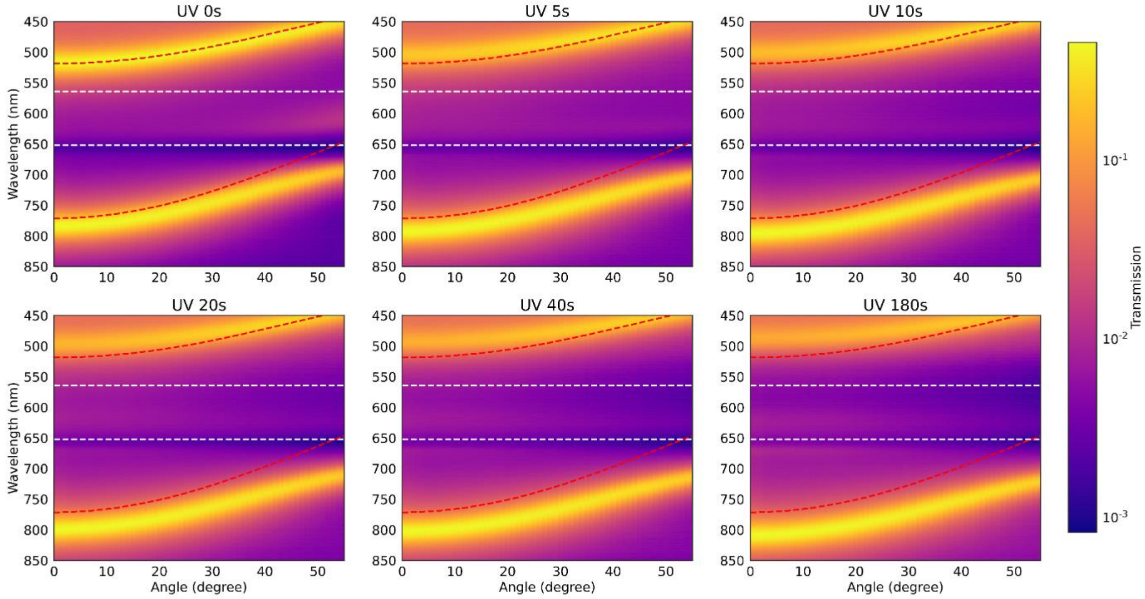

Fig. S32: Experimental angle-resolved transmission maps of the off-resonant cavity at different UV exposure times.

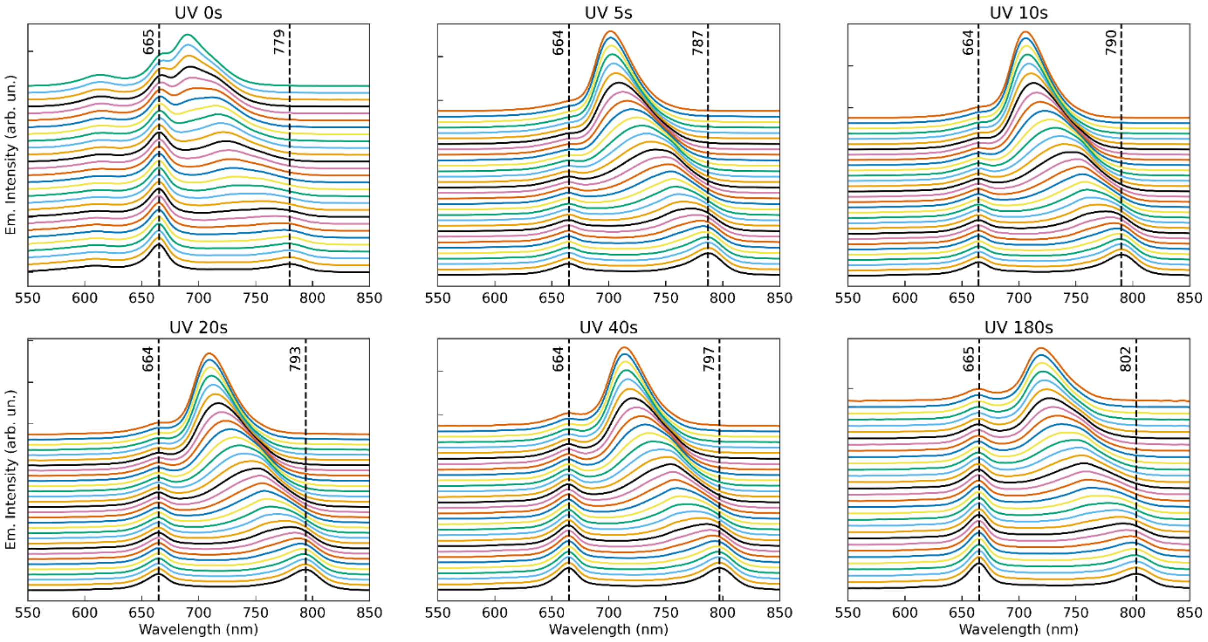

Fig. S33: Experimental angle-resolved emission spectra of the off-resonant cavity as a function of the UV light exposure time. Excitation wavelength: 532 nm. Each spectrum is measured at a given angle value, each with 2° spacing from the previous one, starting from the bottom of the plot. For each UV exposure time, spectra are normalised to the maximum emission intensity of the one measured at 0°. The vertical dashed lines identify the peaks wavelength (nm) at 0° of emission.

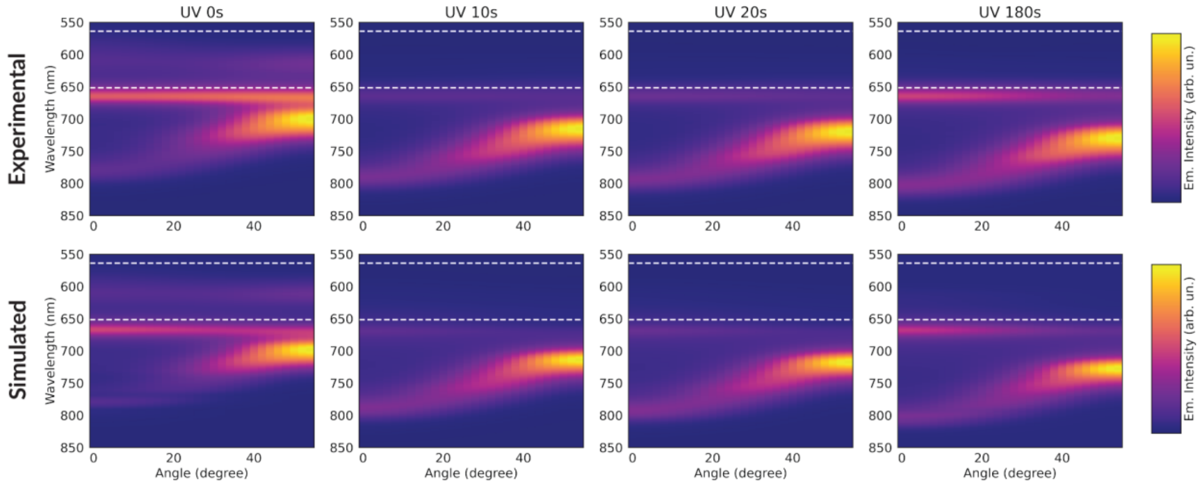

Fig. S34: Angle-resolved PL. Experimental and simulated angle-resolved emission maps of the cavity undergone UV exposure for 0 s, 10 s, 20 s and 180 s (from left to right) and then excited by a 532 nm pump. White dashed lines show the spectral wavelengths of donor and acceptor absorption peaks.

To fit the experimental emission data, we employed the procedure described in the main text. The results of the simulations are presented in the bottom row of Figure S34. The fitting results accurately reproduce the experimental emission maps. The corresponding weight coefficients for these simulations are shown in Figure 6e of the main manuscript. Furthermore (Figure 6d of the main manuscript), using a similar procedure, we determined the weight coefficients corresponding to BRK and SP/MC molecules for the emission from the multilayer outside the cavity (with film thicknesses corresponding to the off-resonant cavity experiment).

The weight coefficients obtained with the off-resonant cavity thicknesses exhibit a similar qualitative behavior over time for both the outside-cavity and inside-cavity cases. Moreover, for the outside-cavity scenario, the contributions of BRK and MC to the emission show similar values to those obtained in the resonant case, which can be attributed to the approximately constant ratio between the thicknesses of the active layers in both experiments.

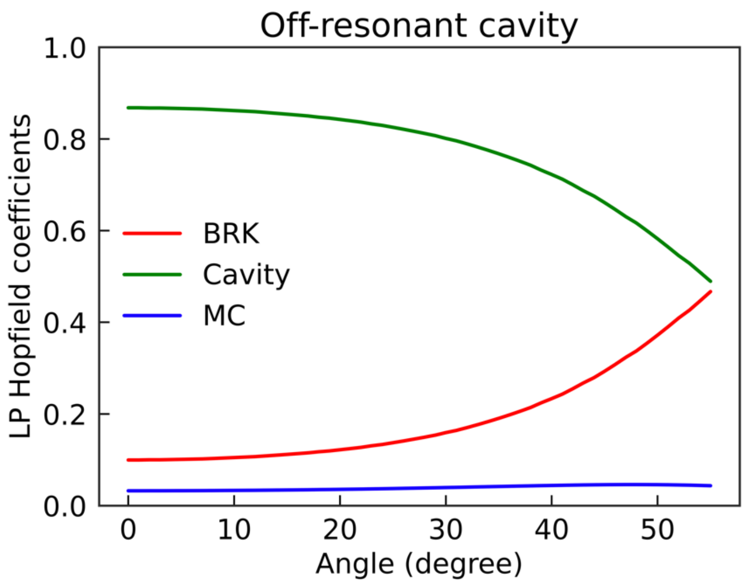

Fig. S35: Hopfield coefficients for the LP branch of the off-resonant cavity.

References

[1] M. J. Schnepf *et al.*, ‘Nanorattles with tailored electric field enhancement’, *Nanoscale*, vol. 9, no. 27, pp. 9376–9385, Jul. 2017, doi: 10.1039/C7NR02952G.

[2] Szczurowski M. Refractiveindex.Info. https://refractiveindex.info/?shelf=organic&book=poly(methyl_methacrylate)&page=Szczurowski.

[3] D. M. Coles *et al.*, ‘Polariton-mediated energy transfer between organic dyes in a strongly coupled optical microcavity’, *Nat. Mater.*, vol. 13, no. 7, Art. no. 7, Jul. 2014, doi: 10.1038/nmat3950.

[4] P. Michetti and G. C. La Rocca, ‘Simulation of J-aggregate microcavity photoluminescence’, *Phys. Rev. B*, vol. 77, no. 19, p. 195301, May 2008, doi: 10.1103/PhysRevB.77.195301.

[5] D. Dovzhenko *et al.*, ‘Polariton-assisted manipulation of energy relaxation pathways: donor–acceptor role reversal in a tuneable microcavity’, *Chem. Sci.*, vol. 12, no. 38, pp. 12794–12805, Oct. 2021, doi: 10.1039/D1SC02026A.

[6] G. Baillet, G. Giusti, and R. Guglielmetti, ‘Comparative photodegradation study between spiro[indoline—oxazine] and spiro[indoline—pyran] derivatives in solution’, *J. Photochem. Photobiol. Chem.*, vol. 70, no. 2, pp. 157–161, Feb. 1993, doi: 10.1016/1010-6030(93)85036-8.

[7] A. Tork, F. Boudreault, M. Roberge, A. M. Ritcey, R. A. Lessard, and T. V. Galstian, ‘Photochromic behavior of spiropyran in polymer matrices’, *Appl. Opt.*, vol. 40, no. 8, pp. 1180–1186, Mar. 2001, doi: 10.1364/AO.40.001180.

[8] R. Matsushima, M. Nishiyama, and M. Doi, ‘Improvements in the fatigue resistances of photochromic compounds’, *J. Photochem. Photobiol. Chem.*, vol. 139, no. 1, pp. 63–69, Feb. 2001, doi: 10.1016/S1010-6030(00)00422-6.

[9] R. Ji *et al.*, ‘Fluorescent Holographic Fringes with a Surface Relief Structure Based on Merocyanine Aggregation Driven by Blue-violet Laser’, *Sci. Rep.*, vol. 8, no. 1, Art. no. 1, Feb. 2018, doi: 10.1038/s41598-018-22202-2.
